# *In Vivo* Real-Time Metabolic Isotopic Tracing via Breath Analysis upon Deuterated Water Ingestion: A Proof-of-Concept Study

**DOI:** 10.1021/acscentsci.6c00078

**Published:** 2026-06-25

**Authors:** Zhihong Yin, Kapil Dev Singh, Zhifeng Tang, Mélina Richard, Jiafa Zeng, Urs Frey, Pablo Sinues, Xue Li

**Affiliations:** † Department of Biomedical Engineering, 27209 University of Basel, Allschwil 4123, Switzerland; ‡ 30280 University Children’s Hospital Basel, Basel 4056, Switzerland; § College of Environment and Climate, Guangdong Provincial Key Laboratory of Speed Capability Research, College of Life Science and Technology, 47885 Jinan University, Guangzhou 510632, China; ∥ Guangdong A-HealthX Technologies Co., Ltd, Dongguan 523830, China; ⊥ The First Affiliated Hospital of Jinan University, Guangzhou 510632, China

## Abstract

Stable isotope tracing with deuterium oxide (D_2_O) offers a powerful approach for probing metabolic dynamics, yet noninvasive and real-time tools for monitoring *in vivo* deuterium (D) incorporation remain scarce. This study introduces secondary electrospray ionization–high-resolution mass spectrometry (SESI-HRMS) as a noninvasive, real-time approach for tracking D incorporation in human exhaled breath metabolites. A total of 11 healthy volunteers were enrolled across two independent study centers. Each participant provided up to 20 breath samples across fasting, post-D_2_O intake, and postprandial phases, yielding 213 samples in total. SESI-HRMS enabled the detection of 2,691 deuterium/hydrogen (D/H) pairs and revealed distinct metabolic trajectories associated with D labeling. Short-chain fatty acids (SCFAs) exhibited biphasic kinetics consistent with rapid orogastric exchange followed by microbial fermentation. Transient increases in pyruvaldehyde were associated with glycemic fluctuations, while dynamic alterations in medium-chain acylcarnitines suggested shifts in mitochondrial energy metabolism. Notably, SESI-HRMS unambiguously resolved D-labeled peaks from natural ^13^C isotopologues, demonstrating high analytical specificity for stable isotope tracing. Collectively, these findings demonstrate the feasibility of SESI-HRMS real-time breath analysis for noninvasive investigation of D-labeled metabolic dynamics in humans.

## Graphical abstract

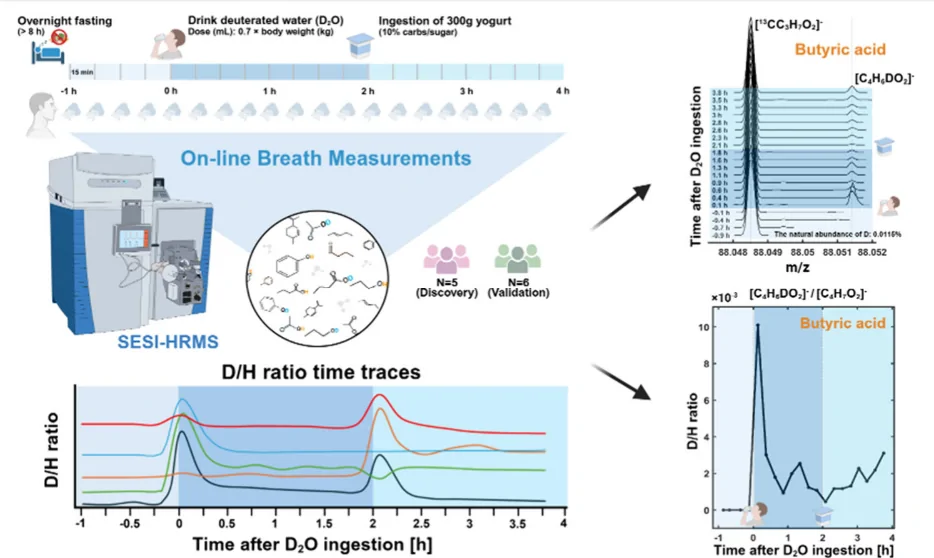

## Introduction

Monitoring biomolecule levels and metabolic fluxes is crucial for elucidating metabolic mechanisms in biological systems and their alterations in response to disease and dietary interventions. Stable isotope tracing techniques excel at this objective, with deuterium (D) standing out due to its global labeling capacity and ease of administration.^1,2^ D can be incorporated into nearly all biomolecules through D–H exchange, thereby enabling safe and cost-effective metabolic labeling. The natural abundance of D is exceedingly low (∼0.0115%),^3^ which minimizes interference from the natural isotopic background and enables highly sensitive detection of D-labeled compounds. Deuterium oxide (D_2_O) introduces D into biological systems directly via oral administration. After ingestion, it is absorbed within 75–120 min and rapidly equilibrates with body water pools, such as plasma, urine, and saliva.^4^ D_2_O labeling has become a powerful tool for metabolic studies, including monitoring lipid^5–9^ and carbohydrate^10^ synthesis and turnover, as well as protein dynamics.^11,12^

Despite its promise, most current implementations of D-based metabolic tracing rely on biofluid or tissue sampling followed by liquid- or gas chromatography–mass spectrometry (LC–MS or GC–MS), limiting their suitability for noninvasive, high-frequency, or real-time monitoring. More recently, deuterium metabolic imaging (DMI) has further highlighted the broader potential of D-based metabolic interrogation *in vivo* by combining D-labeled substrates with magnetic resonance spectroscopic imaging to generate spatially resolved metabolic maps within tissues.^3^ In parallel, real-time breath analysis has emerged as a compelling noninvasive approach for monitoring metabolic dynamics, with exhaled volatile and semivolatile metabolites providing access to systemic metabolic processes.^13^ Within the broader landscape, breath-based isotopic tracing may complement DMI by offering a distinct type of metabolic readout. Whereas DMI emphasizes spatially resolved mapping of labeled metabolism within tissues, breath analysis is well suited for real-time and repeated monitoring of dynamic metabolic changes reflected in exhaled metabolites. Isotope-based breath studies have already shown considerable clinical potential, as exemplified by the widely used ^13^C-urea breath test for *Helicobacter pylori* diagnosis.^14^ However, breath-based D_2_O labeling for metabolite-resolved monitoring of metabolic dynamics remains largely unexplored.

Pioneering work using flowing afterglow mass spectrometry (FA-MS) has demonstrated that D_2_O can enable real-time and noninvasive tracking of total body water *in vivo* through exhaled breath analysis.^15–17^ However, the limited selectivity and low resolution of FA-MS prevent the detection of trace-level metabolites in breath and the resolution of complex molecular mixtures,^18^ posing a key barrier to advancing D-based breathomics. Building on our prior animal study,^19^ here we extend D-resolved secondary electrospray ionization–high-resolution mass spectrometry (SESI-HRMS)^20^ for real-time and noninvasive isotopic tracing of breath metabolites following D_2_O ingestion in humans across fasting and postprandial states.

## Results and Discussion

A total of 11 healthy volunteers were enrolled across two study centers. In the discovery cohort, 5 healthy volunteers (3 males and 2 females; mean age, 35.2 ± 10.8 years) were recruited in Switzerland from July to September 2022. The validation cohort consisted of 6 participants (5 males and 1 female; mean age, 25.3 ± 5.7 years), who were recruited in China in September 2023. Participant characteristics, including age, sex, weight, body mass index (BMI), and D_2_O dosing, are shown in Table 1. All participants performed 20 breath measurements (4 pre- and 16 post-D_2_O ingestion), except for one subject, who provided 13 measurements (1 pre- and 12 post-D_2_O ingestion), yielding a total of 213 breath measurements.

**Table 1:** Participant Characteristics

**Group/Subject No.**	**Sex**	**Age (years)**	**Weight (kg)**	**BMI (kg/m** ^**2**^ **)**	**Dose D**_**2**_ **O (mL)**
**Group 1: Discovery cohort - Switzerland (CH)**					
CH-1	M	34	77.8	27.57	54.46
CH-2	M	46	71.5	22.32	50.05
CH-3	F	40	61	21.11	42.70
CH-4	M	29	62.8	21.23	43.96
CH-5	F	27	58	20.55	40.60
**Group 2: Validation cohort - China (CN)**					
CN-1	M	26	52	17.99	36.40
CN-2	M	31	88	29.75	61.60
CN-3	M	24	69	20.60	48.30
CN-4	M	24	61	18.83	42.70
CN-5	M	23	64	23.51	44.80
CN-6	F	24	50	20.28	35.00

Upon preprocessing the raw mass spectra, we generated a data matrix of 213 × 6,305 (samples × *m*/*z* features). Figure 1A shows an example set of 20 breath mass spectra from a representative participant in the *m*/*z* region 88.0473–88.0521, corresponding to the M + 1 peak region of deprotonated butyric acid.^21,22^ Consistent with our previous animal study,^19^ we detected a distinct D incorporation peak that appeared after D_2_O ingestion, whose relative intensity (normalized to the corresponding ^13^C isotopic peak) varied over time. This observation underscored the robustness and sensitivity of our approach for detecting even minute D incorporation, and the dynamic changes in peak intensity may reflect ongoing metabolic processes *in vivo*. Notably, the high resolution of SESI-HRMS enabled the unambiguous separation of D-labeled peaks from the naturally occurring ^13^C isotopic peak,^8^ allowing true D incorporation to be distinguished from ^13^C isotopic interference.

**Figure 1:**
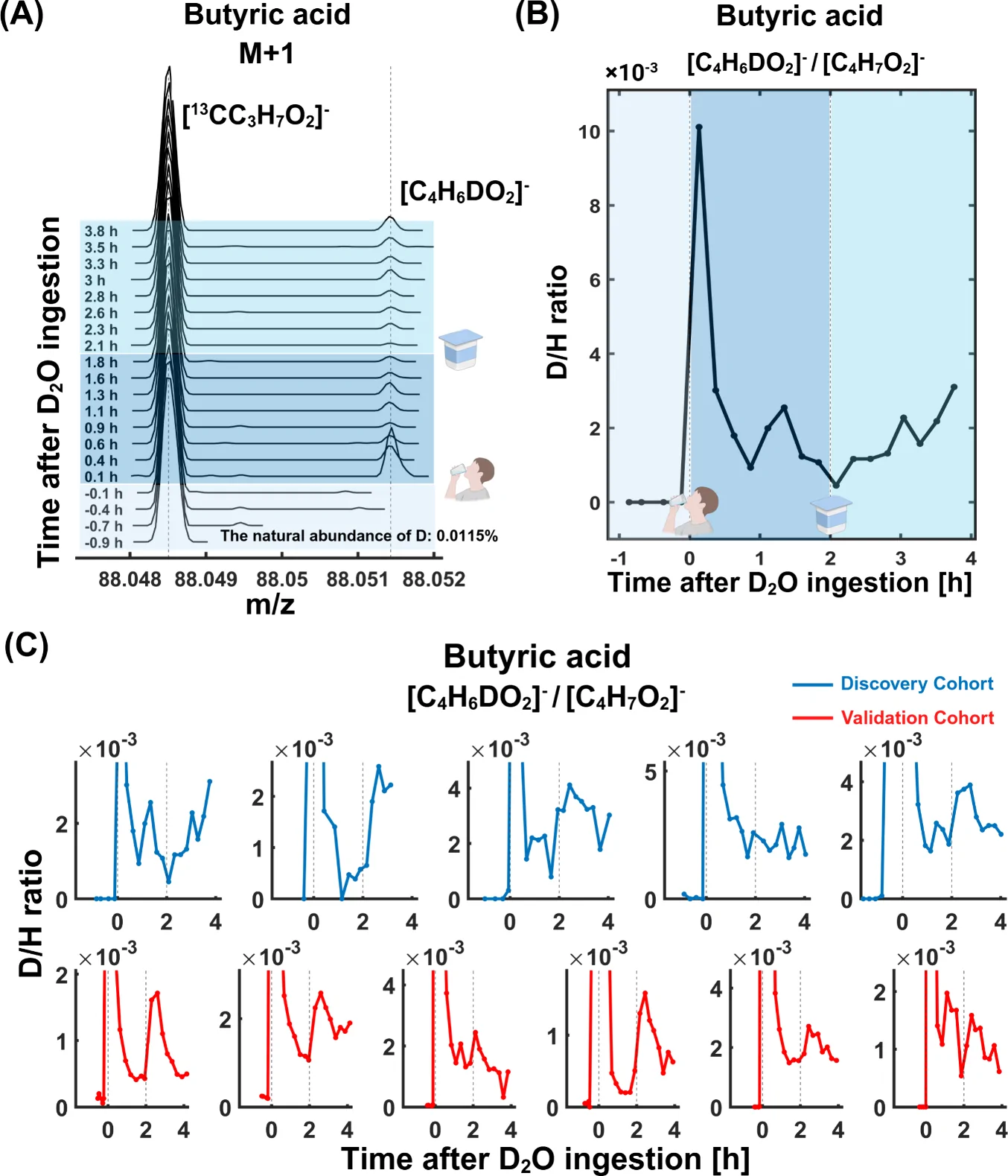
Real-time tracking of D incorporation in exhaled butyric acid. (A) High-resolution M + 1 mass spectrum of exhaled deprotonated butyric acid [C_4_H_7_O_2_]^−^ from a representative subject, demonstrating ^13^C and D-labeled peaks. Experimental peak positions align closely with exact *m*/*z* values (dashed lines). (B) Temporal profile of the deprotonated butyric acid D/H ratio for the same participant as in panel A. Dashed lines indicate the time points of D_2_O and yogurt ingestion. (C) Individual D/H ratio temporal profiles of deprotonated butyric acid across the study cohort (*N* = 11). The *y*-axis is adjusted to 0–1.2 × *Y*_3_ (where *Y*_3_ denotes the third-highest value) to improve visualization of the time-course changes; the full-scale profiles are shown in Figure S1. Blue lines represent the discovery cohort (*N* = 5), and red lines represent the validation cohort (*N* = 6). H = hydrogen; D = deuterium; D_2_O = deuterium oxide.

Building on these observations, we next examined the temporal profile of the isotopic ratio ([C_4_H_6_DO_2_]^−^/[C_4_H_7_O_2_]^−^) of deprotonated butyric acid. Figure 1B shows the corresponding time trace from the same participant as in Figure 1A. The ratio remained at background levels during the four data points preceding D_2_O ingestion. Immediately after D_2_O ingestion, the ratio peaked within 5 min and declined by nearly 70% at 15 min. Given that complete absorption of orally ingested D_2_O and its equilibration with body fluids generally require approximately 75–120 min, this rapid decrease most likely reflects clearance of residual D_2_O in the oral cavity.^4,16^ This characteristic “spike-then-drop” pattern was consistently observed across all individuals in both cohorts (Figure S1). At the group level (Figure 1C), the overall temporal pattern remained broadly similar across individuals. Over the two hours after D_2_O ingestion, the D/H ratio rose rapidly, followed by a pronounced decline and subsequent stabilization. After yogurt consumption, the ratio increased again among most participants. Some interindividual variability was observed at baseline, which may partly reflect differences in fasting status. Participants were instructed to fast for at least eight hours, but fasting duration beyond this minimum was not further specified. In addition, subtle differences were also observed across individuals in the magnitude and temporal profile of the later response. Given that short-chain fatty acids (SCFAs) originate primarily from gut microbial metabolism,^23,24^ these differences may reflect variation in gut microbial composition and metabolic activity among participants. Although yogurt intake was standardized to 300 g and the products showed broadly comparable macronutrient profiles (approximately 10% carbohydrates, 3% protein, and 10% fat, predominantly saturated), the yogurt used in the two cohorts was purchased locally. As a result, compositional differences, including microbial content, may have contributed to the variability observed between cohorts.

To systematically characterize the dynamic profiles of D-enriched molecular features across fasting and feeding states, we identified isotopologue pairs differing by *n* × 1.0063 Da (i.e., mass difference between H and D, where *n* = 1, 2, 3, ...) and calculated their signal intensity ratios. This procedure yielded 2,691 isotopic D/H pairs, including 2,372 detected in positive ion mode and 319 in negative ion mode. Based on the BIC profile, which reached a plateau at approximately K = 10 (Figure S2), noise-enhanced von Mises–Fisher mixture (NAvMix)^25^ clustering identified ten temporal trajectory clusters comprising 1,026 isotopologue pairs (Figures S3 and S4), each cluster representing a group of isotopologue pairs that shared a similar response profile across the experimental time course. We first selected positive response clusters showing increased D-labeling signals during the fasting and/or postprandial phases. The dominant temporal characteristics of these selected clusters are summarized in Table S1. Among these, we focused subsequent analyses on three representative clusters (Clusters #4, #7, and #10, 315 isotopologue pairs in total), which captured distinct positive response patterns (Figure 2A): a biphasic response spanning the post-D_2_O and postprandial phases (Cluster #4), a sustained positive postprandial increase (Cluster #7), and a sharp transient postprandial surge (Cluster #10). The 315 isotopologue pairs assigned to these three representative clusters were subsequently mapped to the Human Metabolome Database (HMDB),^26^ and the corresponding putative annotations are provided in Supplementary Table 1.

**Figure 2:**
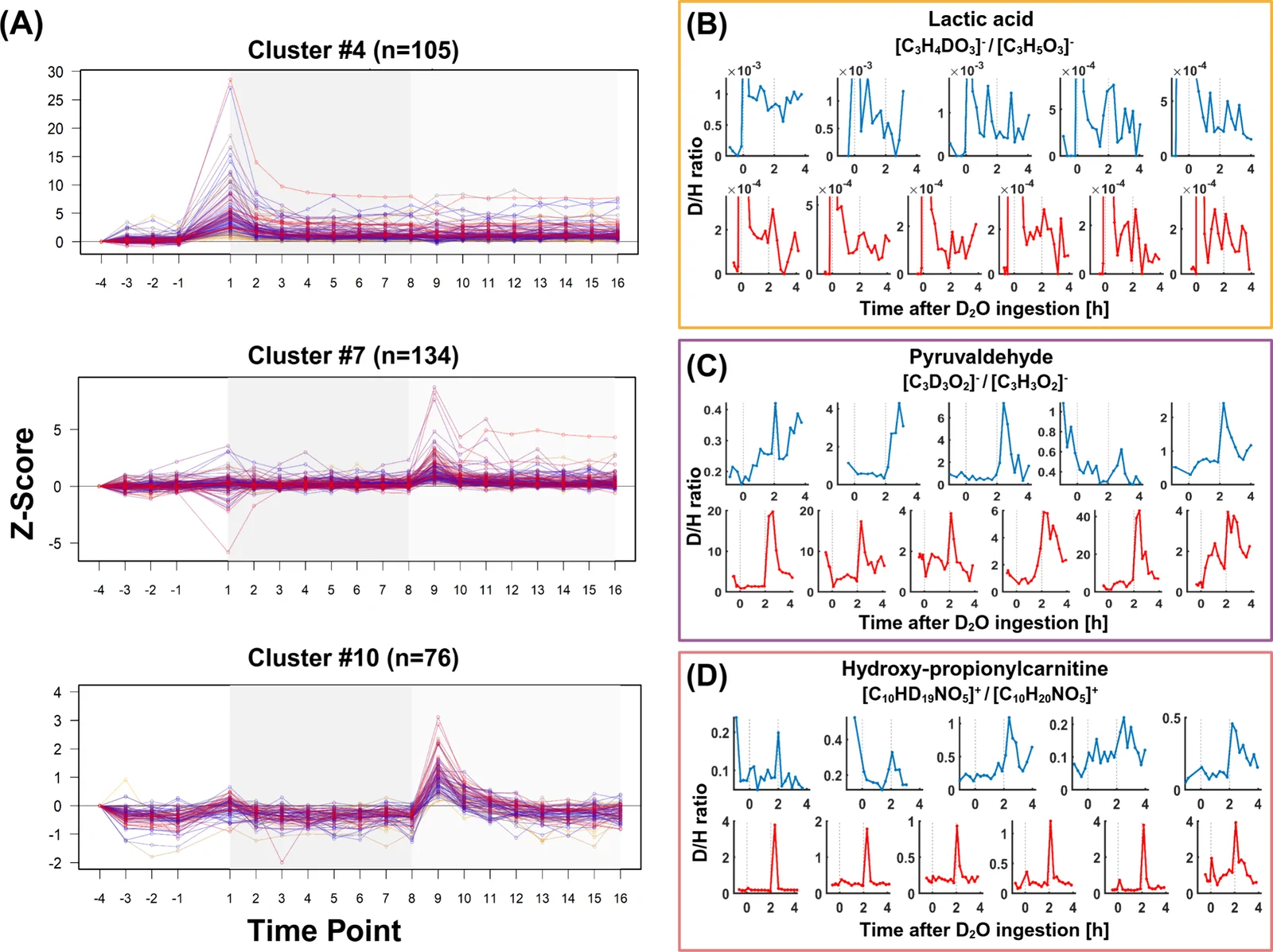
Cluster-based temporal profiles of D/H ratios following D_2_O and yogurt intake. (A) Standardized mean D/H ratio trajectories for three representative positive response clusters selected from the ten-cluster NAvMix model. Each panel represents the standardized mean D/H ratio of all isotopologue pairs within the corresponding cluster, normalized by the mean and standard deviation of the −4 time point. (B–D) Representative D/H ratio temporal profiles for putatively annotated features characteristic of each cluster: deprotonated lactic acid [C_3_H_5_O_3_]^−^ for Cluster #4 (B), deprotonated pyruvaldehyde [C_3_H_3_O_2_]^−^ for Cluster #7 (C), and protonated hydroxy-propionylcarnitine [C_10_H_20_NO_5_]^+^ for Cluster #10 (D). Blue lines denote the discovery cohort (*N* = 5), and red lines denote the validation cohort (*N* = 6).

Cluster #4 (*n* = 105) represented the biphasic response spanning the post-D_2_O and postprandial phases. Although its temporal profile resembled that of Cluster #5, Cluster #4 maintained D/H ratios above baseline after the initial peak, whereas Cluster #5 returned more closely toward baseline (Figure 2A and Figure S4). This more sustained post-D_2_O response motivated our focus on Cluster #4 for subsequent analyses. The D/H ratio increased immediately following D_2_O intake, declined rapidly, and rose again approximately 1–1.5 h after yogurt ingestion. Putative metabolite annotations (Supplementary Table 1) suggested enrichment of SCFA-related features, including propionic, valeric/isovaleric and caproic acids, together with other small organic acids such as lactic acid (Figure 2B). This compositional bias was further supported by RefMet-based enrichment analysis,^27^ which identified a significant over representation of oxygenated hydrocarbons (Fisher’s exact test, *p* < 0.001; Figure S5). The shared biphasic temporal pattern observed across these putatively annotated metabolites suggests a common underlying source or coordinated response. The initial spike likely reflects transient local D–H exchange with residual D_2_O in the oral and upper gastrointestinal compartments before systemic equilibration. This interpretation is also consistent with previously reported early deuterium kinetics in saliva and blood following D_2_O ingestion.^28–30^ The delayed postprandial increase is compatible with microbial fermentation of dietary carbohydrates following food intake, a process known to produce lactic acid and SCFAs.^23^ For example, increased carbon availability has been shown to shift bifidobacterial metabolism toward acetate and lactic acid production.^23^

Cluster #7 (*n* = 134) displayed a sustained postprandial response following yogurt ingestion (Figure 2A). The D/H ratio remained near baseline during the baseline and early post-D_2_O phases, aside from a modest transient increase after D_2_O ingestion. After yogurt ingestion, the ratio rose to a pronounced postprandial peak, then declined and stabilized above baseline over the subsequent two hours. Putative annotations suggested that this cluster involved features matching several chemical classes, including prenol lipids, organooxygen compounds, fatty acyls, carboxylic acids and derivatives, together accounting for over half of the assigned features (68/134; Supplementary Table 1). Consistent with this chemical diversity, RefMet-based enrichment analysis did not identify any single chemical class reaching statistical significance (Figure S6), indicating substantial chemical heterogeneity within this cluster. Pyruvaldehyde provides an illustrative example at the level of an individual putatively annotated feature (Figure 2C). Pyruvaldehyde, also known as methylglyoxal, has been described as a glycolytic byproduct formed from glycerol-3-phosphate (G3P) and dihydroxyacetone phosphate (DHAP),^31^ and is normally counteracted by the glyoxalase system.^32^ This biochemical context may be compatible with the observed peak-decline pattern, although direct metabolite confirmation was not performed in the present study. Other putatively annotated features in this cluster included γ-aminobutyric acid (GABA), malondialdehyde, dimethylglycine, β-aminobutyric acids, and pyroglutamic acid. These candidate annotations are associated with processes such as protein intake, lipid peroxidation, one-carbon metabolism, amino acid catabolism, and glutathione turnover, respectively. In a broader physiological context,^31,33^ these findings highlight the potential of D_2_O-based breath analysis to capture dynamic changes across chemically diverse metabolic features, pending further targeted validation.

Cluster #10 (*n* = 76) shared a postprandial-dominant trajectory with Cluster #7 but exhibited a more pronounced postprandial response, characterized by a shaper surge immediately after yogurt ingestion followed by a decline toward baseline (Figure 2A). Putative annotations (Supplementary Table 1) and RefMet-based enrichment analysis suggested an over representation of short-chain acylcarnitine-related features in this cluster (Figure S7; *p* = 0.001), predominantly C_3_–C_6_ species, including oxo- and hydroxy-propionylcarnitines, as well as butyryl-, valeryl-, and hexanoyl-carnitines. Hydroxy-propionylcarnitine provides an illustrative example of this within-cluster trajectory (Figure 2D). Acylcarnitines have been widely associated with mitochondrial fuel handling and metabolic flexibility during fasting-to-feeding transitions.^34–36^ In this context, the pronounced postprandial response observed here may be compatible with short-term changes in mitochondrial acyl-group handling as substrate availability shifts after yogurt ingestion. The predominance of C_3_–C_6_ acylcarnitine-related features may reflect contributions from multiple metabolic inputs, including branched-chain amino acid (BCAA) catabolism and gut microbial SCFA metabolism.^34–38^

To directly compare isotopic incorporation across clusters, Figure 3A summarizes the distributions in the apparent number of exchanged D atoms (*n*). Cluster #4 was dominated by single-site D incorporation, with 86% of features (90/105) assigned to *n* = 1. In comparison, Clusters #7 and #10 showed broader multisite labeling distributions, with the most frequent assignment at *n* = 3, accounting for 36% of features in Cluster #7 (48/134) and 43% in Cluster #10 (33/76). Notably, Cluster #10 also contained a distinct high-D-incorporation subgroup, with a prominent peak at *n* = 19 (Figure 3A), driven predominantly by acylcarnitine-associated features. Among the 16 carnitine-assigned features in cluster #10 (Supplementary Table 1), 12 were observed at *n* = 19, whereas the remaining four were detected at *n* = 13 (1 feature), *n* = 11 (1 feature), and *n* = 7 (2 features). To assess the reliability of the dominant 19 D assignments, we selected eight putative 19D-labeled acylcarnitine features with sufficient signal quality for isotopic pattern validation. For these eight features (Table S2), measured isotopic patterns showed close agreement with simulated patterns in both the ^12^C and corresponding ^13^C windows (Figure 3B; Figure S8). Pearson correlation analysis across breath samples further supported these assignments, with median r values of 0.956 for the ^12^C window, 0.858 for the ^13^C window, and 0.958 for the combined fit (Figure 3C). Mechanistically, the high D counts observed for acylcarnitine-related features may be with multisite incorporation of water-derived hydrogens into acyl-CoA and carnitine metabolism through β-oxidation associated reactions and NAD­(P)­H-dependent reductions. For a C_10_ acyl moiety, four β-oxidation cycles would involve approximately 16–20 potentially labelable positions, consistent with the *n* ≈ 19 isotopologue observed here.^39,40^ By contrast, Cluster #4 was characterized mainly by single-site D–H exchange (∼86% 1-D incorporation), consistent with its enrichment in organic acids and rapid, nonenzymatic D–H exchange of carboxylic OH groups. Together, these comparisons help distinguish shallow exchange from multisite metabolic incorporation and suggest that D_2_O-based labeling may capture distinct patterns of postprandial metabolic turnover across chemically and physiologically diverse metabolite pools.

**Figure 3:**
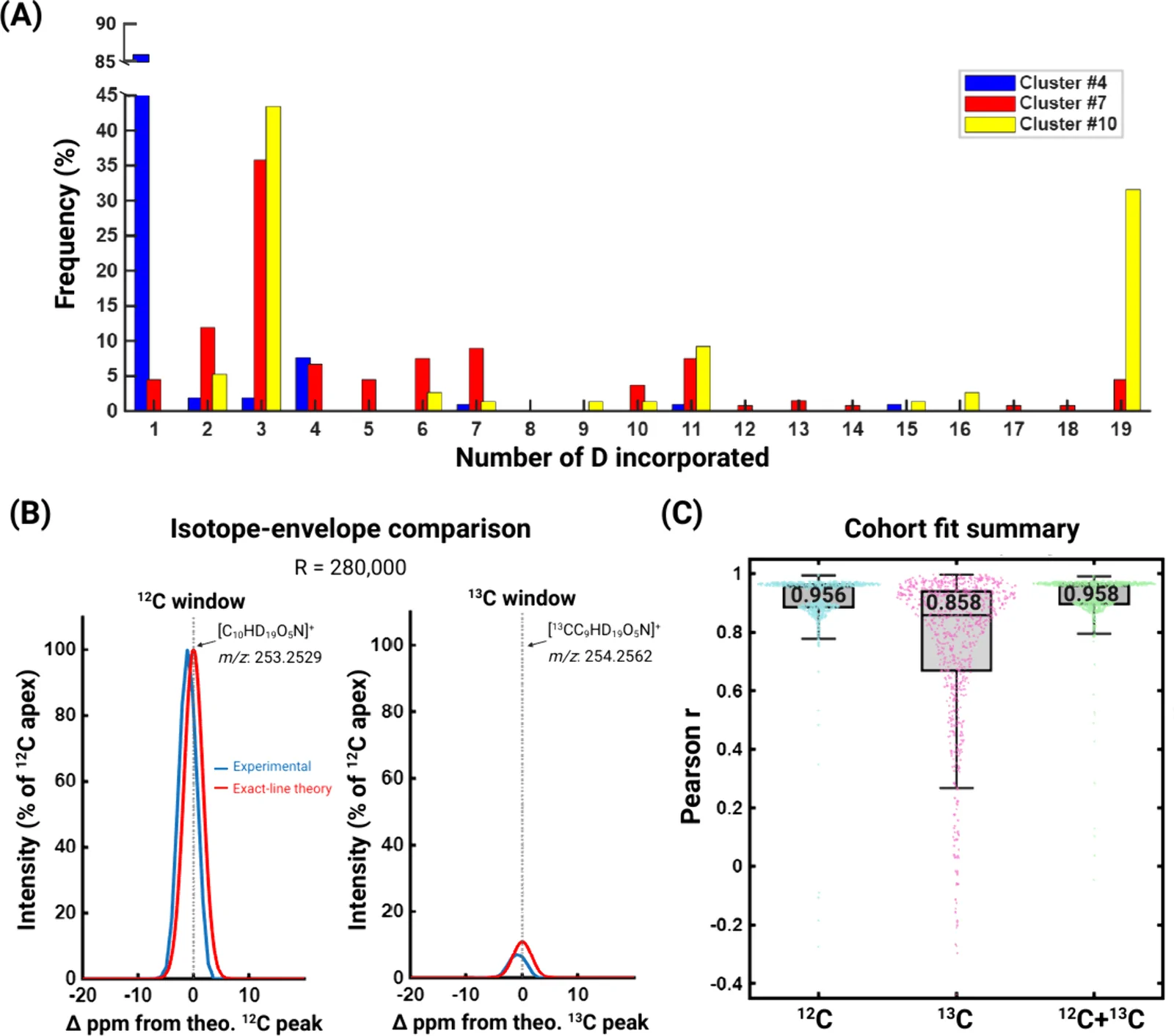
Deuterium incorporation patterns and isotope-pattern validation of high-level D-incorporation features. (A) Distribution of the apparent number of incorporated deuterium atoms (*n*) across three representative time-course clusters (Clusters #4, #7, and #10). Histograms represent the fraction of features within each cluster that exhibit a given level of D incorporation. The *y*-axis is truncated (45–85%) to improve visualization of differences among lower-frequency incorporation levels. (B) Isotope-envelope comparison for one putative 19D-labeled acylcarnitine ([C_10_HD_19_O_5_N]^+^; *m*/*z*: 253.2529) in a representative breath sample. Experimental profiles are compared with exact-line theoretical profiles simulated at a resolution of 280,000 for the ^12^C and corresponding ^13^C windows, with intensities normalized to the ^12^C apex. Additional putative 19D-labeled acylcarnitine comparisons are shown in Figure S8. (C) Pearson correlation analysis comparing measured and theoretical isotope patterns for the eight selected putative 19D-labeled acylcarnitine features across breath samples. Only samples in which both the ^12^C and corresponding ^13^C centroids were detected within ± 5 ppm of the theoretical *m*/*z* values were considered evaluable.

To obtain a systematic view of the biology captured by D_2_O-based isotopic labeling combined with real-time SESI-HRMS breath analysis, we performed pathway enrichment analysis (Figure 4). Out of a total of 2,691 isotopic D/H pairs, 315 isotopologue pairs assigned to Clusters #4, #7, and #10 were selected as the significant set, with the remaining pairs used as background for pathway enrichment analysis based on Fisher’s exact test. The analysis revealed significant over-representation (*p* < 0.05) of pathways associated with central carbon and amino-acid metabolism,^41^ including glycine/serine/threonine metabolism, butanoate metabolism, valine/leucine/isoleucine biosynthesis and degradation, propanoate metabolism, and pyruvate metabolism (Figure 4; Supplementary Table 2; Figure S9). At the network level, these enriched pathways appeared to converge on a limited number of highly connected metabolic hubs, including pyruvate metabolism and SCFA-related pathways. This convergence suggests that breath metabolomics may capture coordinated, system-level metabolic changes across the organism, rather than merely isolated biochemical reactions. Upon intake, D_2_O rapidly equilibrates with the body-water hydrogen pool, enabling solvent-mediated D–H exchange at labile sites and incorporation into newly formed C–H (C–D) bonds during enzyme-catalyzed reactions.^42–44^ Pathways with high turnover and abundant exchangeable or transferable hydrogens may therefore be more susceptible to D labeling and may be detected earlier in D_2_O-based tracing readouts. The observed enrichment of pyruvate/lactate-related metabolites is consistent with rapid turnover of the pyruvate–lactate nodes and its dependence on NADH-mediated redox cycling, which differs substantially between fasting and postprandial states.^45–47^ Through one-carbon metabolism, D tracers in serine may be transferred into NAD­(P)H and subsequently into malate, proline, and other downstream metabolites.^48–50^ BCAA catabolism supplies carbon to acetyl-CoA and succinyl-CoA.^51^ BCAAs, their α-keto acids, and succinate-linked tricarboxylic acid (TCA) intermediates have been associated with differential substrate utilization across metabolic states.^51–55^ SCFAs are key microbiota-derived fuels that influence host energy homeostasis and signaling.^56,57^ Fasting promotes hepatic ketogenesis (β-hydroxybutyrate and acetoacetate), whereas feeding shifts metabolism toward carbohydrate utilization.^56^ Accordingly, changes in D_2_O labeling of SCFA-related features may reflect the combined effects of host fuel switching and the availability and oxidation of microbiota-derived SCFAs.^56–58^

**Figure 4:**
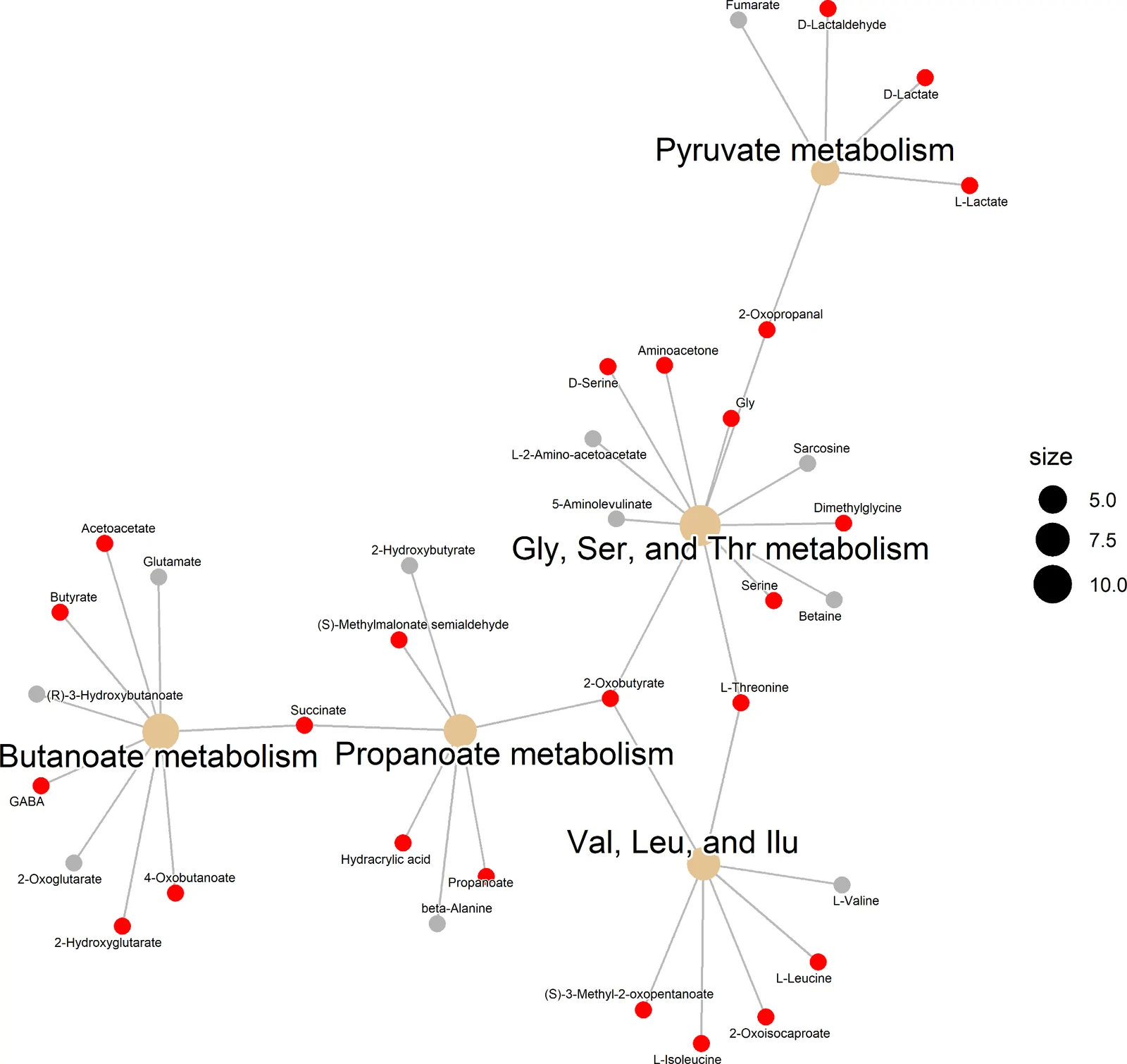
Network view of enriched metabolic pathways and mapped metabolites. The bigger, tan-colored nodes denote metabolic pathways identified as significantly enriched by Fisher’s exact test using isotopologue pairs from Clusters #4, #7, and #10 as the significant set and all remaining pairs as background. Pathway node size reflects the number of cluster-associated isotopic features mapped to each pathway. Remaining smaller nodes represent mapped metabolites. Red circles indicate compounds assigned to the isotopologue pairs belonging to Clusters #4, #7, or #10, whereas gray circles represent compounds assigned to the background features. Enrichment statistics and the ranked pathways are provided in Figure S9 and Supplementary Table 2.

While this study demonstrates the utility of SESI-HRMS for real-time *in vivo* tracing of D-labeled metabolites after D_2_O administration, several limitations warrant consideration. First, the present study should be regarded as exploratory and hypothesis-generating, given the modest sample size (N = 11) and restriction to healthy young volunteers. These factors constrain statistical power for broader biological inference and limit the generalizability of our findings to broader and clinically relevant populations. Future work should therefore evaluate this approach in larger, more heterogeneous cohorts, and further refine its application across diverse complementary experimental systems. Second, our experimental protocol captured metabolic responses over a relatively short 5-h fasting-to-feeding window, which constrains the assessment of longer-term metabolic adaptations. Extending breath sampling over longer periods in future studies could enable characterization of slower kinetic processes and provide a clearer view of interindividual variability in D_2_O incorporation dynamics. Third, metabolite annotation in the current work remains putative because it relies primarily on accurate-mass MS^1^ data, while interpretations involving potential microbial contributions are further limited by the absence of direct microbiome characterization. Future studies combining multiomics readouts (e.g., microbiome profiling) with systematic MS/MS validation, and confirmation with chemical standards for key metabolites, should improve annotation confidence and mechanistic insight.^50,54^ Expanding breath-metabolite libraries via community standards will further enhance translational readiness.^50,59^

## Conclusions

In this proof-of-concept study, we show that D_2_O-based isotopic labeling combined with SESI-HRMS enables noninvasive, time-resolved assessment of metabolic dynamics through exhaled breath. High-resolution isotopic analysis revealed reproducible labeling dynamics across fasting-to-feeding transitions and linked D–H exchange features to central carbon, SCFA, and amino-acid metabolism. Together, these findings support breath-based deuterium tracing as a dynamic *in vivo* readout of nutritional and metabolic responses, with particular relevance for postprandial metabolic phenotyping, metabolic flexibility, and host-microbial metabolic interactions. With further targeted validation in larger and more diverse cohorts, this framework may provide a useful tool for investigating metabolic conditions marked by impaired postprandial control, altered carbon fluxes, or dysregulated lipid handling, particularly insulin resistance and early type 2 diabetes, and potentially related obesity-associated metabolic dysfunction.

## Supplementary Material









## Notes

Figures of D/H-ratio time-trace profiles, NAvMix clustering analyses, heatmap of cluster of D/H ratio time traces behavior, time traces of 1,026 standardized D/H ratios, pathway enrichment analyses, and targeted exact-line isotope validation with isotopic-envelope comparisons for putative highly labeled acylcarnitine features; tables of selected NAvMix clusters and theoretical *m*/*z* values; experimental section (PDF)
Supplementary Table 1: Putative HMDB annotations for 315 isotopologue pairs from Clusters #4, #7, and #10 (CSV)
Supplementary Table 2: Pathway enrichment statistics for Clusters #4, #7, and #10 isotopologue pairs against the remaining pairs (CSV)
Transparent Peer Review report available (PDF)

## References

[1] Kim J., Seo S., Kim T. Y. (2023). Metabolic deuterium oxide (D2O) labeling in quantitative
omics studies: A tutorial review. Anal. Chim.
Acta.

[2] Miyagi M., Kasumov T. (2016). Monitoring the synthesis
of biomolecules using mass
spectrometry. Philos Trans. R. Soc. A.

[3] De
Feyter H. M., Behar K. L., Corbin Z. A., Fulbright R. K., Brown P. B., McIntyre S., Nixon T. W., Rothman D. L., de Graaf R. A. (2018). Deuterium metabolic imaging (DMI) for MRI-based 3D
mapping of metabolism in vivo. Sci. Adv..

[4] Peronnet F., Mignault D., du Souich P., Vergne S., Le Bellego L., Jimenez L., Rabasa-Lhoret R. (2012). Pharmacokinetic
analysis of absorption,
distribution and disappearance of ingested water labeled with D2O
in humans. Eur. J. Appl. Physiol..

[5] Leitch C. A., Jones P. J. (1993). Measurement of human lipogenesis using deuterium incorporation. J. Lipid Res..

[6] Diraison F., Pachiaudi C., Beylot M. (1996). In vivo measurement of plasma cholesterol
and fatty acid synthesis with deuterated water: Determination of the
average number of deuterium atoms incorporated. Metabolism.

[7] Strawford A., Antelo F., Christiansen M., Hellerstein M. K. (2004). Adipose
tissue triglyceride turnover, de novo lipogenesis, and cell proliferation
in humans measured with 2H2O. Am. J. Physiol.:
Endocrinol. Metab..

[8] Fu X. R., Deja S., Fletcher J. A., Anderson N. N., Mizerska M., Vale G., Browning J. D., Horton J. D., McDonald J. G., Mitsche M. A., Burgess S. C. (2021). Measurement
of lipogenic flux by
deuterium resolved mass spectrometry. Nat. Commun..

[9] Kostyukevich Y., Stekolshikova E., Levashova A., Kovalenko A., Vishnevskaya A., Bashilov A., Kireev A., Tupertsev B., Rumiantseva L., Khaitovich P. (2023). Untargeted Lipidomics after D2O Administration
Reveals the Turnover Rate of Individual Lipids in Various Organs of
Living Organisms. Int. J. Mol. Sci..

[10] Schumann W. C., Gastaldelli A., Chandramouli V., Previs S. F., Pettiti M., Ferrannini E., Landau B. R. (2001). Determination of the Enrichment of
the Hydrogen Bound to Carbon 5 of Glucose on 2H2O Administration. Anal. Biochem..

[11] Wilkinson D. J., Franchi M. V., Brook M. S., Narici M. V., Williams J. P., Mitchell W. K., Szewczyk N. J., Greenhaff P. L., Atherton P. J., Smith K. (2014). A validation of the
application of
D2O stable isotope tracer techniques for monitoring day-to-day changes
in muscle protein subfraction synthesis in humans. Am. J. Physiol. Endocrinol. Metab..

[12] Price J. C., Holmes W. E., Li K. W., Floreani N. A., Neese R. A., Turner S. M., Hellerstein M. K. (2012). Measurement
of human plasma proteome
dynamics with 2H2O and liquid chromatography tandem mass spectrometry. Anal. Biochem..

[13] Popov T. A. (2011). Human exhaled
breath analysis. Ann. Allergy, Asthma, Immunol..

[14] Savarino V., Vigneri S., Celle G. (1999). The 13C urea breath test in the diagnosis
ofHelicobacter pylori infection. Gut.

[15] Chan C., Smith D., Spanel P., McIntyre C. W., Davies S. J. (2008). A non-invasive,
on-line deuterium dilution technique for the measurement of total
body water in haemodialysis patients. Nephrol.
Dial. Transplant..

[16] Davies S., Spanel P., Smith D. (2001). Rapid measurement
of deuterium content
of breath following oral ingestion to determine body water. Physiol. Meas..

[17] Smith D., Engel B., Diskin A. M., Španěl P., Davies S. J. (2002). Comparative measurements of total body water in healthy
volunteers by online breath deuterium measurement and other near-subject
methods2. Am. J. Clin. Nutr..

[18] Smith D., Španěl P. (2015). SIFT-MS and
FA-MS methods for ambient gas phase analysis:
developments and applications in the UK. Analyst.

[19] Arnold K., Chen X., Zhang H., Singh K. D., Yin Z., Yao Y., Luan T., Sinues P., Li X. (2022). In vivo detection of
metabolic 2H-incorporation upon ingestion of 2H2O. J. Bio-x Res..

[20] Gisler A., Singh K. D., Zeng J., Osswald M., Awchi M., Decrue F., Schmidt F., Sievi N., Chen X., Usemann J. (2022). An Interoperability
Framework for Multicentric Breath
Metabolomic Studies. iScience.

[21] Adam M. E., Fehervari M., Boshier P. R., Chin S.-T., Lin G.-P., Romano A., Kumar S., Hanna G. B. (2019). Mass-Spectrometry
Analysis of Mixed-Breath, Isolated-Bronchial-Breath, and Gastric-Endoluminal-Air
Volatile Fatty Acids in Esophagogastric Cancer. Anal. Chem..

[22] Martinez-Lozano P., Fernandez de la Mora J. (2008). Direct analysis of
fatty acid vapors in breath by electrospray
ionization and atmospheric pressure ionization-mass spectrometry. Anal. Chem..

[23] Macfarlane S., Macfarlane G. T. (2003). Regulation
of short-chain fatty acid production. Proc.
Nutr. Soc..

[24] Dalile B., Van Oudenhove L., Vervliet B., Verbeke K. (2019). The role of short-chain
fatty acids in microbiota-gut-brain communication. Nat. Rev. Gastroenterol. Hepatol..

[25] Grant A. J., Gill D., Kirk P. D. W., Burgess S. (2022). Noise-augmented directional
clustering of genetic association data identifies distinct mechanisms
underlying obesity. PLoS Genet..

[26] Wishart D. S., Guo A., Oler E., Wang F., Anjum A., Peters H., Dizon R., Sayeeda Z., Tian S., Lee B. L. (2022). HMDB
5.0: the Human Metabolome Database for 2022. Nucleic Acids Res..

[27] Fahy E., Subramaniam S. (2020). RefMet: a
reference nomenclature for metabolomics. Nat.
Methods.

[28] Huttenhower C., Gevers D., Knight R., Abubucker S., Badger J. H., Chinwalla A. T., Creasy H. H., Earl A. M., FitzGerald M. G., Fulton R. S. (2012). Structure, function
and diversity of the healthy human microbiome. Nature.

[29] Gao L., Xu T., Huang G., Jiang S., Gu Y., Chen F. (2018). Oral microbiomes:
more and more importance in oral cavity and whole body. Protein & Cell.

[30] Jankowski C. M., Sonko B. J., Gozansky W. S., Kohrt W. M. (2004). Deuterium Dilution:
The Time Course of 2H Enrichment in Saliva, Urine, and Serum. Clin. Chem..

[31] Bellier J., Nokin M.-J., Lardé E., Karoyan P., Peulen O., Castronovo V., Bellahcène A. (2019). Methylglyoxal, a potent inducer of
AGEs, connects between diabetes and cancer. Diabetes Res. Clin. Pract..

[32] Rabbani N., Thornalley P. J. (2012). Methylglyoxal, glyoxalase 1 and the
dicarbonyl proteome. Amino Acids.

[33] Nokin M.-J., Bellier J., Durieux F., Peulen O., Rademaker G., Gabriel M., Monseur C., Charloteaux B., Verbeke L., van Laere S. (2019). Methylglyoxal, a glycolysis
metabolite, triggers metastasis through MEK/ERK/SMAD1 pathway activation
in breast cancer. Breast Cancer Res..

[34] Gojda J., Cahova M. (2021). Gut Microbiota as the
Link between Elevated BCAA Serum
Levels and Insulin Resistance. Biomolecules.

[35] Li T.-T., Chen X., Huo D., Arifuzzaman M., Qiao S., Jin W.-B., Shi H., Li X. V., Iliev I. D., Artis D., Guo C.-J. (2024). Microbiota
metabolism
of intestinal amino acids impacts host nutrient homeostasis and physiology. Cell Host Microbe.

[36] Yoshida N., Yamashita T., Osone T., Hosooka T., Shinohara M., Kitahama S., Sasaki K., Sasaki D., Yoneshiro T., Suzuki T. (2021). Bacteroides spp. promotes branched-chain amino acid
catabolism in brown fat and inhibits obesity. iScience.

[37] Newgard C. B., An J., Bain J. R., Muehlbauer M. J., Stevens R. D., Lien L. F., Haqq A. M., Shah S. H., Arlotto M., Slentz C. A. (2009). A Branched-Chain Amino Acid-Related Metabolic Signature that Differentiates
Obese and Lean Humans and Contributes to Insulin Resistance. Cell Metab..

[38] Dhakan D. B., Maji A., Sharma A. K., Saxena R., Pulikkan J., Grace T., Gomez A., Scaria J., Amato K. R., Sharma V. K. (2019). The unique composition of Indian
gut microbiome, gene
catalogue, and associated fecal metabolome deciphered using multi-omics
approaches. GigaScience.

[39] Houten S. M., Violante S., Ventura F. V., Wanders R. J. A. (2016). The Biochemistry
and Physiology of Mitochondrial Fatty Acid β-Oxidation and Its
Genetic Disorders. Annu. Rev. Physiol..

[40] Turner S. M., Murphy E. J., Neese R. A., Antelo F., Thomas T., Agarwal A., Go C., Hellerstein M. K. (2003). Measurement
of TG synthesis and turnover in vivo by 2H2O incorporation into the
glycerol moiety and application of MIDA. Am.
J. Physiol.: Endocrinol. Metab..

[41] Kambhampati S., Hubbard A. H., Koley S., Gomez J. D., Marsolais F. d. r., Evans B. S., Young J. D., Allen D. K. (2024). SIMPEL: using stable
isotopes to elucidate dynamics of context specific metabolism. Commun. Biol..

[42] Damont A., Legrand A., Cao C., Fenaille F., Tabet J.-C. (2023). Hydrogen/deuterium
exchange mass spectrometry in the world of small molecules. Mass Spectrom. Rev..

[43] Zhang Z., Chen L., Liu L., Su X., Rabinowitz J. D. (2017). Chemical
Basis for Deuterium Labeling of Fat and NADPH. J. Am. Chem. Soc..

[44] Smith D. A., Nakamoto B. J., Suess M. K., Fogel M. L. (2022). Central Metabolism
and Growth Rate Impacts on Hydrogen and Carbon Isotope Fractionation
During Amino Acid Synthesis in E. coli. Front.
Microbiol..

[45] Corkey B. E., Deeney J. T. (2020). The Redox Communication
Network as a Regulator of Metabolism. Front.
Physiol..

[46] Bartman C. R., Faubert B., Rabinowitz J. D., DeBerardinis R. J. (2023). Metabolic
pathway analysis using stable isotopes in patients with cancer. Nat. Rev. Cancer.

[47] Xu J., Martien J., Gilbertson C., Ma J., Amador-Noguez D., Park J. O. (2020). Metabolic flux analysis
and fluxomics-driven determination
of reaction free energy using multiple isotopes. Curr. Opin. Biotechnol..

[48] Ducker G. S., Rabinowitz J. D. (2017). One-Carbon Metabolism in Health and
Disease. Cell Metab..

[49] Yang Y., Fan T. W. M., Lane A. N., Higashi R. M. (2017). Chloroformate derivatization
for tracing the fate of Amino acids in cells and tissues by multiple
stable isotope resolved metabolomics (mSIRM). Anal. Chim. Acta.

[50] Tejero
Rioseras A., Singh K. D., Nowak N., Gaugg M. T., Bruderer T., Zenobi R., Sinues P. M. L. (2018). Real-Time Monitoring
of Tricarboxylic Acid Metabolites in Exhaled Breath. Anal. Chem..

[51] Neinast M., Murashige D., Arany Z. (2019). Branched Chain Amino Acids. Annu. Rev. Physiol..

[52] Holeček M. (2020). Why Are Branched-Chain
Amino Acids Increased in Starvation and Diabetes?. Nutrients.

[53] Cagigas M. L., Santiappillai N. T., Commissati S., Fiorito G., Masedunskas A., Rajakumar G., de Ciutiis I., Goldhamer A., Kennedy B. K., Hoy A. J., Fontana L. (2025). The Metabolic Transition
Between Fasting and Feeding Alters Aging-Associated Metabolites, Lowers
BCAAs, and Stimulates FGF21 Production in Humans. Aging Cell.

[54] Wang L., Xing X., Zeng X., Jackson S. R., TeSlaa T., Al-Dalahmah O., Samarah L. Z., Goodwin K., Yang L., McReynolds M. R. (2022). Spatially resolved isotope tracing reveals
tissue metabolic activity. Nat. Methods.

[55] Stern A., Fokra M., Sarvin B., Alrahem A. A., Lee W. D., Aizenshtein E., Sarvin N., Shlomi T. (2023). Inferring mitochondrial
and cytosolic metabolism by coupling isotope tracing and deconvolution. Nat. Commun..

[56] Hu J., Lin S., Zheng B., Cheung P. C. K. (2018). Short-chain fatty
acids in control
of energy metabolism. Crit. Rev. Food Sci. Nutr..

[57] Kasubuchi M., Hasegawa S., Hiramatsu T., Ichimura A., Kimura I. (2015). Dietary Gut
Microbial Metabolites, Short-chain Fatty Acids, and Host Metabolic
Regulation. Nutrients.

[58] Du Y., He C., An Y., Huang Y., Zhang H., Fu W., Wang M., Shan Z., Xie J., Yang Y., Zhao B. (2024). The Role of
Short Chain Fatty Acids in Inflammation and Body Health. Int. J. Mol. Sci..

[59] Choueiry F., Xu R., Gold A., Jung H., Zhu J. (2025). Online monitoring and
stable isotope tracing of cancer associated volatiles in murine model
captures tumor associated markers in vivo. Anal.
Chim. Acta.

