# Supplementary material for: In Vivo Real-Time Metabolic Isotopic Tracing via Breath Analysis upon Deuterated Water Ingestion: A Proof-of-Concept Study
Source: ACS Cent Sci. 2026 Jun 25;12(8):1110–8. doi: 10.1021/acscentsci.6c00078 (PMC13523499; doi:10.1021/acscentsci.6c00078)
Supplement: Supplementary file 1 [file oc6c00078_si_001.pdf]

Supplemental Information

for

In Vivo Real-Time Metabolic Isotopic Tracing via Breath Analysis upon Deuterated Water Ingestion: A Proof-of-Concept Study

Zhihong Yin[1,2], Kapil Dev Singh[1,2], Zhifeng Tang[3,4], Mélina Richard[1,2], Jiafa Zeng[3,5], Urs Frey[1,2], Pablo Sinues[1,2], Xue Li[3*]

[1] Department of Biomedical Engineering, University of Basel, Allschwil 4123, Switzerland

[2] University Children's Hospital Basel, Basel 4056, Switzerland

[3] College of Environment and Climate, Guangdong Provincial Key Laboratory of Speed Capability Research, College of Life Science and Technology, Jinan University, Guangzhou 510632, China

[4] Guangdong A-HealthX Technologies Co., Ltd, Dongguan 523830, China

[5] The First Affiliated Hospital of Jinan University, Guangzhou 510632, China

*Email: tamylee@jnu.edu.cn

Contents

Section 1: Supplemental Tables and Figures 3

Section 2: Experimental Section 12

Participants 12

Study Design 12

On-line Breath Measurement 13

Instrument Setting and Quality Control 14

Data Analysis and Statistics 14

Putative Metabolite Annotation Strategy 15

Targeted Exact-line Isotope Validation 16

References 17

Section 1： Supplemental Tables and Figures

Figure S1 Full-scale temporal profiles of the deprotonated butyric acid D/H ratio across the study cohort.

Figure S2 Model selection and final cluster sizes for NAvMix clustering of D/H ratio time traces.

Figure S3 Heatmap of D/H ratio time-course profiles across ten NAvMix clusters.

Figure S4 Time-course trajectories of D/H ratios across ten NAvMix clusters.

Table S1 Summary of selected NAvMix clusters exhibiting positive response patterns after D_2O and/or yogurt ingestion

Figure S5 Pathway enrichment analysis of features from Cluster #4 using the RefMet database.

Figure S6 Pathway enrichment analysis of features from Cluster #7 using the RefMet database.

Figure S7 Pathway enrichment analysis of features from Cluster #10 using the RefMet database.

Table S2 Theoretical *m/z* values of monoisotopic and single-^{13}C $[M+H]^+$ peaks and expected $^{13}C/^{12}C$ intensity ratios for the eight putative 19D-labeled acylcarnitines.

Figure S8 Isotopic-envelope comparisons for eight putative 19D-labeled acylcarnitines.

Figure S9 Pathway enrichment analysis of features from Clusters #4, #7, and #10 using the RefMet database

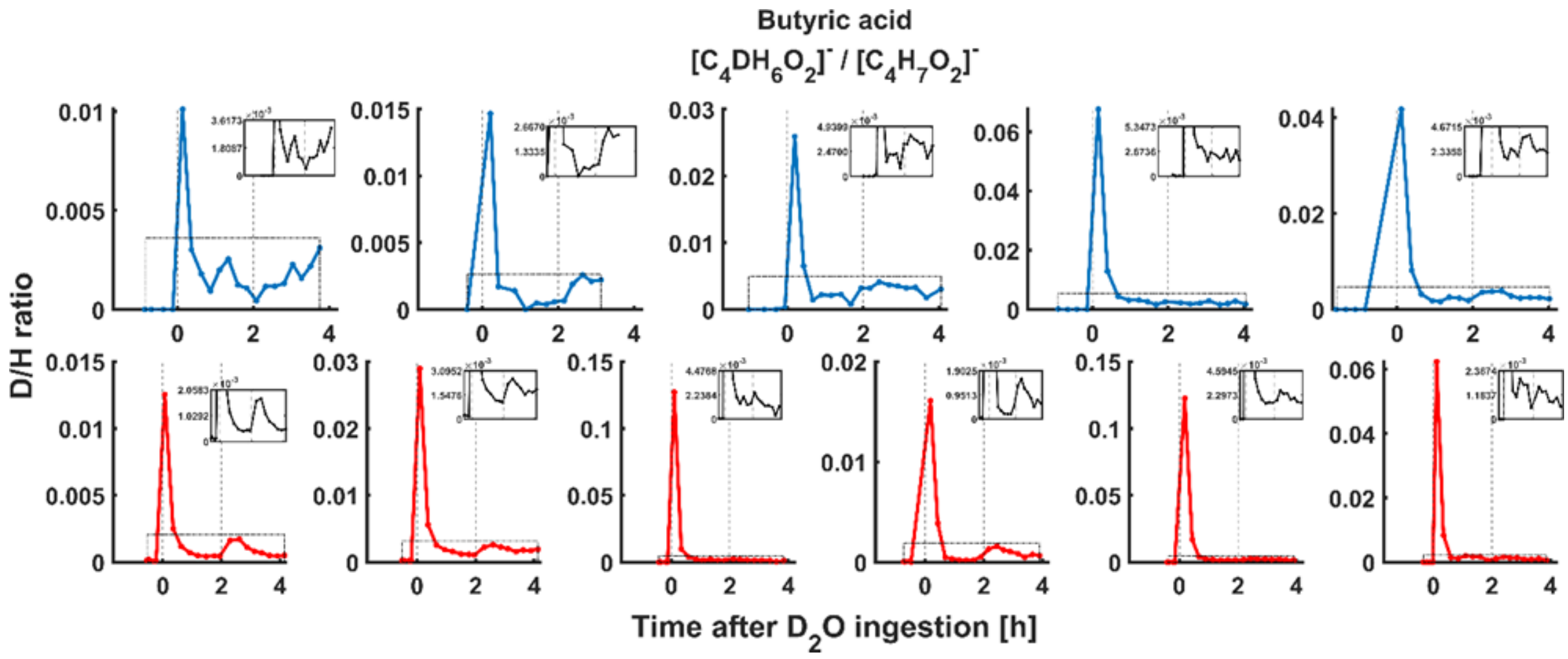

Figure S1 Full-scale temporal profiles of the deprotonated butyric acid D/H ratio across the study cohort. The black dashed rectangles correspond to the y-axis-adjusted regions shown in Figure 1C in the main text and magnified in the upper-right inset of each panel. Blue lines represent the discovery cohort (N = 5), and red lines represent the validation cohort (N = 6). H = hydrogen; D = deuterium; D_2O = deuterium oxide.

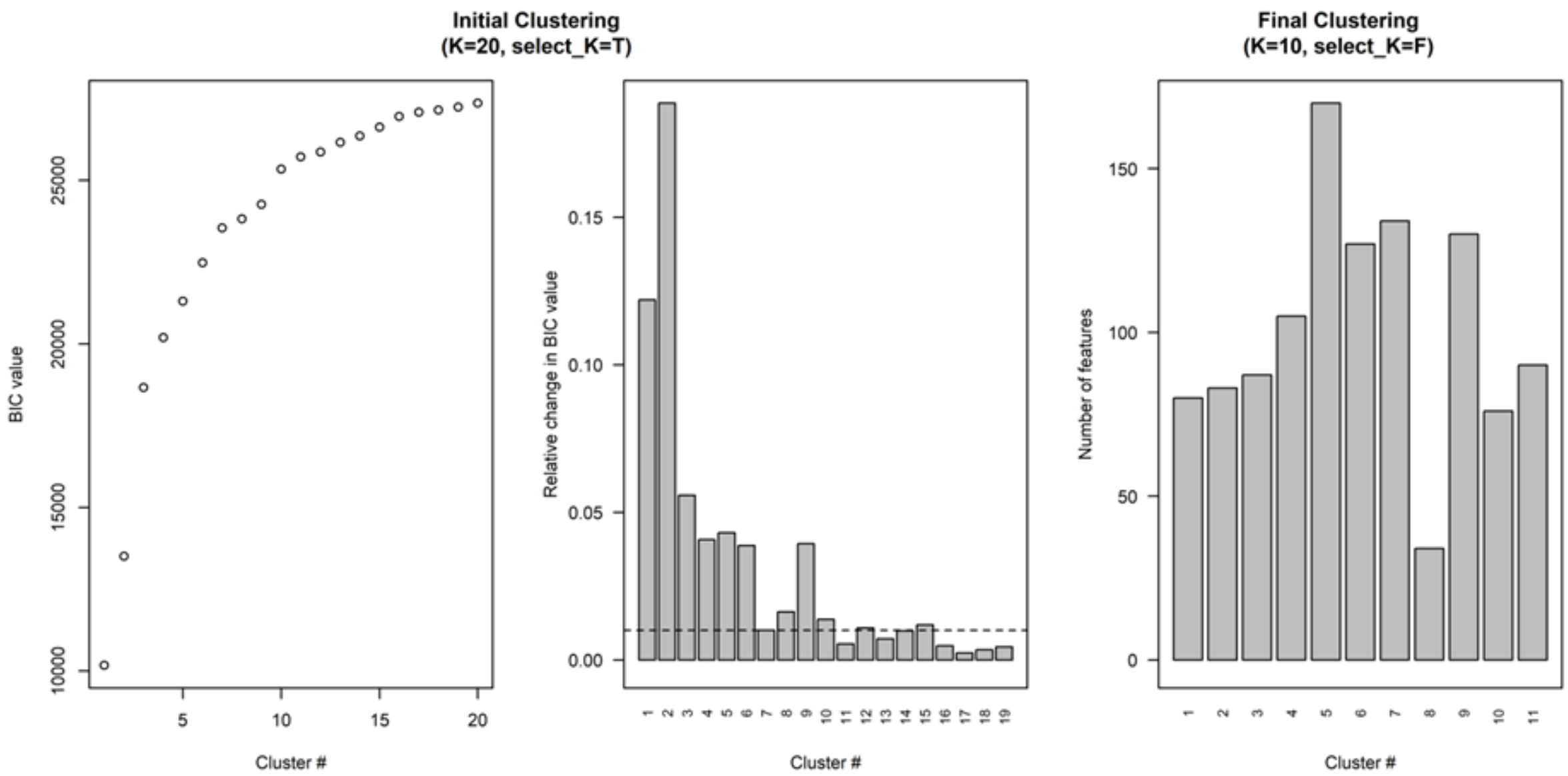

Figure S2 Model selection and final cluster sizes for NAvMix clustering of D/H ratio time traces. NAvMix clustering was applied to 2,691 isotopologue D/H pairs. **Left**: Bayesian information criterion (BIC) across candidate solutions with 1–20 clusters from the initial run (K = 20, select_K = TRUE). **Middle**: Relative change in BIC between successive K values; the dashed line denotes a heuristic threshold below which additional clusters yielded negligible

improvement in model fit, indicating the onset of a plateau. The plateau observed after approximately 10 clusters motivated the selection of K = 10 for the final model. **Right**: Number of features assigned to each cluster in the final model (K = 10, select_K = FALSE).

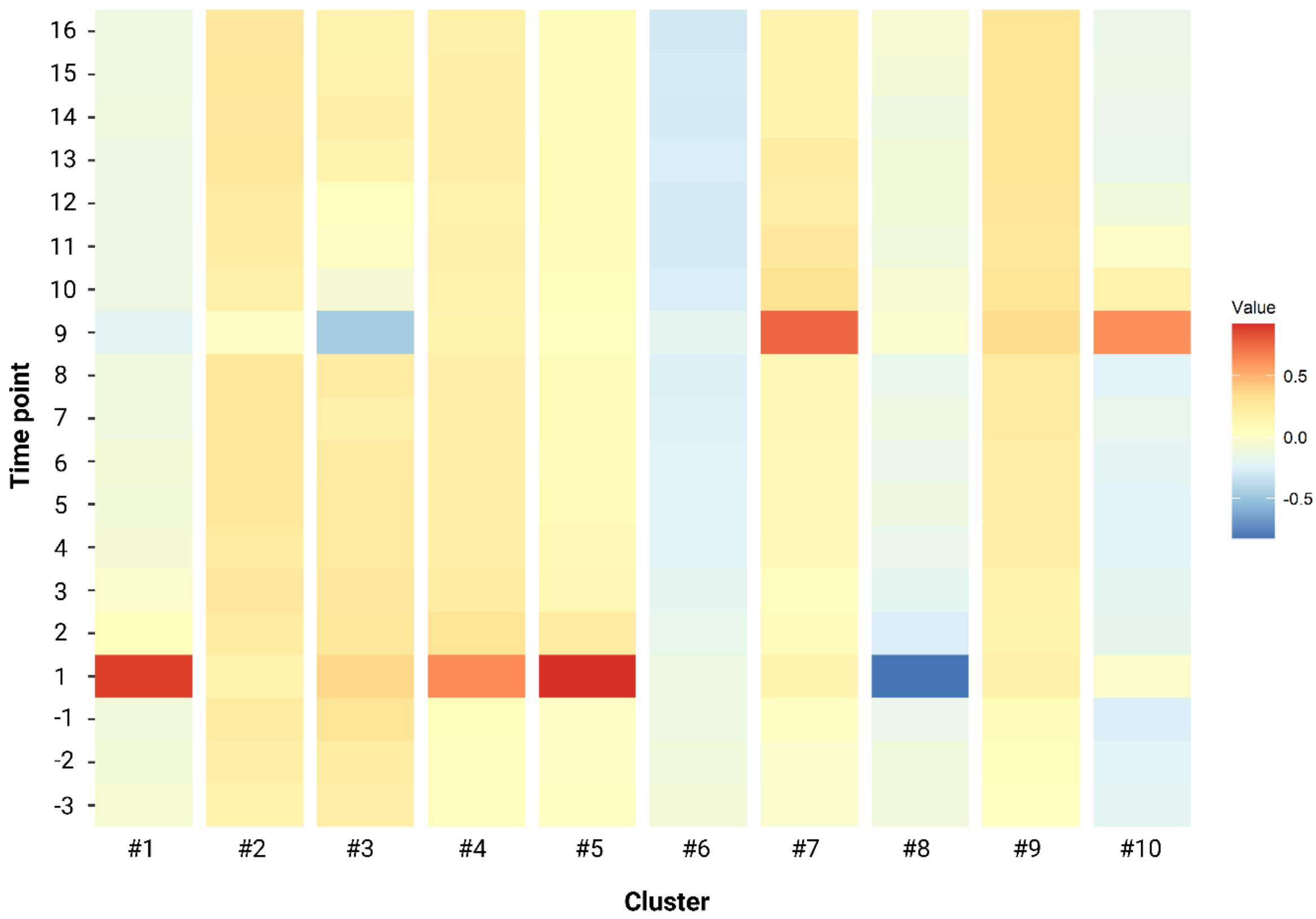

Figure S3 Heatmap of D/H ratio time-course profiles across ten NAvMix clusters. The heat map shows the standardized intensity (z-score) of the D/H ratio at each time point, spanning 3 pre-D_2O ingestion baseline measurements and 16 post-D_2O ingestion measurements at 15-min intervals. Standardization was performed using the mean and standard deviation of the first sample of the baseline. Time point 1 represents the first breath sample collected after D_2O ingestion, and time point 9 represents the first breath sample collected after yogurt consumption. Columns represent the ten NAvMix clusters (#1–#10); warmer colors indicate increases and cooler colors indicate decreases relative to baseline.

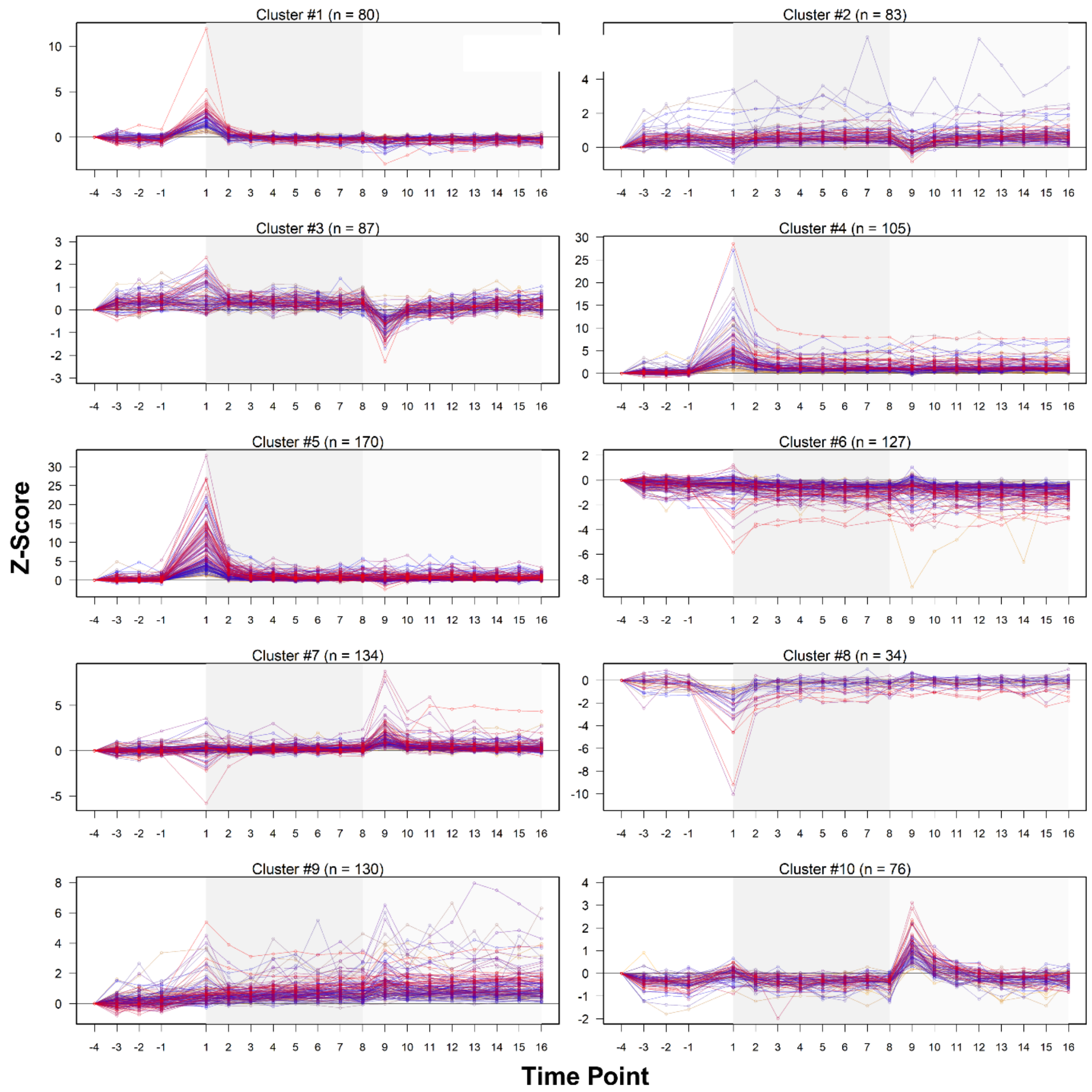

Figure S4 Time-course trajectories of D/H ratios across ten NAvMix clusters. NAvMix clustering was applied to trajectories from 2,691 isotopologue D/H pairs, of which 1,026 were assigned to ten structured clusters; the remaining features were assigned to the noise cluster. Each panel shows all D/H isotopologue-pair trajectories assigned to the corresponding cluster, with each trajectory composed of standardized D/H ratios across breath-sampling time points; the number of features is indicated in the panel title. Standardization was performed using the mean and standard deviation at time point −4. Time point 1 represents the first breath sample collected after D_2O ingestion, and time point 9 represents the first breath sample collected after yogurt consumption; the gray shading indicates the interval between D_2O ingestion and yogurt consumption.

Table S1 Summary of selected NAvMix clusters exhibiting positive response patterns after D_2O and/or yogurt ingestion

Cluster	n	Dominant temporal characteristic	Role in interpretation
1	80	Immediate sharp post-D_2O spike followed by a rapid return toward baseline	Not prioritized; less representative of sustained labeling dynamics
4	105	Sustained early positive response after D_2O administration	Prioritized as a biphasic response spanning the post-D_2O and postprandial phases
5	170	Very strong immediate post-D_2O spike followed by rapid return toward baseline	Not prioritized; less representative of sustained labeling dynamics
7	134	Sustained positive postprandial increase	Prioritized as a representative sustained postprandial pattern
9	130	Modest but sustained positive postprandial trend	Considered but not prioritized, because the trajectory was less distinct
10	76	Sharp transient postprandial surge followed by decline	Prioritized as a representative transient postprandial pattern

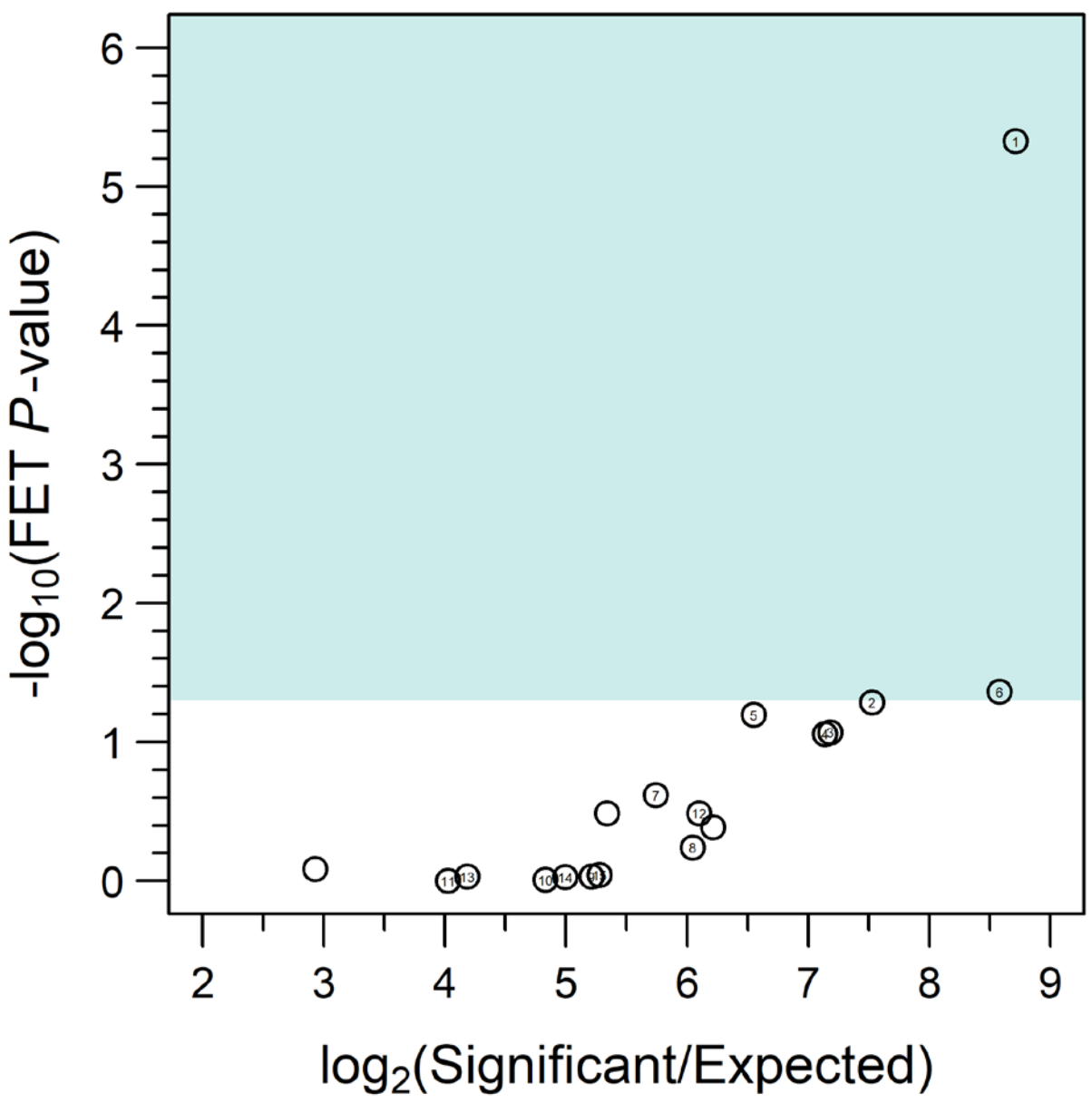

	Pathways	Size	Matches: Total	Matches: Significant	Matches: Expected	P-values: FET	P-values: EASE	P-values: Γ
1	Oxygenated hydrocarbons	17	11	8	0.02	4.7e-06	6.9e-05	0.091
2	Fatty aldehydes	29	23	6	0.03	0.052	0.14	0.098
3	Oxo Fatty Acids	43	32	7	0.05	0.085	0.18	0.100
4	Branched Fatty Acids	38	26	6	0.04	0.087	0.20	0.10
5	Saturated Fatty Acids	38	13	4	0.04	0.064	0.20	0.10
6	Fatty lactones	7	7	3	0.01	0.043	0.20	0.10
7	Fatty alcohols	50	14	3	0.06	0.24	0.52	0.12
8	C10 isoprenoids	81	49	6	0.09	0.57	0.73	0.15
9	Unsaturated Fatty Acids	265	125	11	0.30	0.93	0.96	0.23
10	C15 isoprenoids	94	58	3	0.11	0.98	1.0	0.31
11	Hydroxy Fatty Acids	274	112	5	0.31	1.0	1.0	0.42
12	Monoalkylamines	13	3	1	0.01	0.33	1.0	1.0
13	Dicarboxylic acids	49	20	1	0.05	0.93	1.0	1.0
14	Phenylpropanoic acids	28	21	1	0.03	0.94	1.0	1.0
15	Amino Fatty Acids	23	18	1	0.03	0.91	1.0	1.0

Figure S5 Pathway enrichment analysis of features from Cluster #4 using the RefMet database. Pathway enrichment was evaluated using Fisher's exact test (FET). The significant set comprised 105 isotopologue D/H pairs assigned to Cluster #4, selected from a total of 2,691 pairs; the remaining pairs were used as the background set. The left panel shows a scatter plot of enriched pathways, with the shaded region indicating significantly enriched pathways ($p < 0.05$). The right panel lists the top 15 enriched pathways ranked by statistical significance.

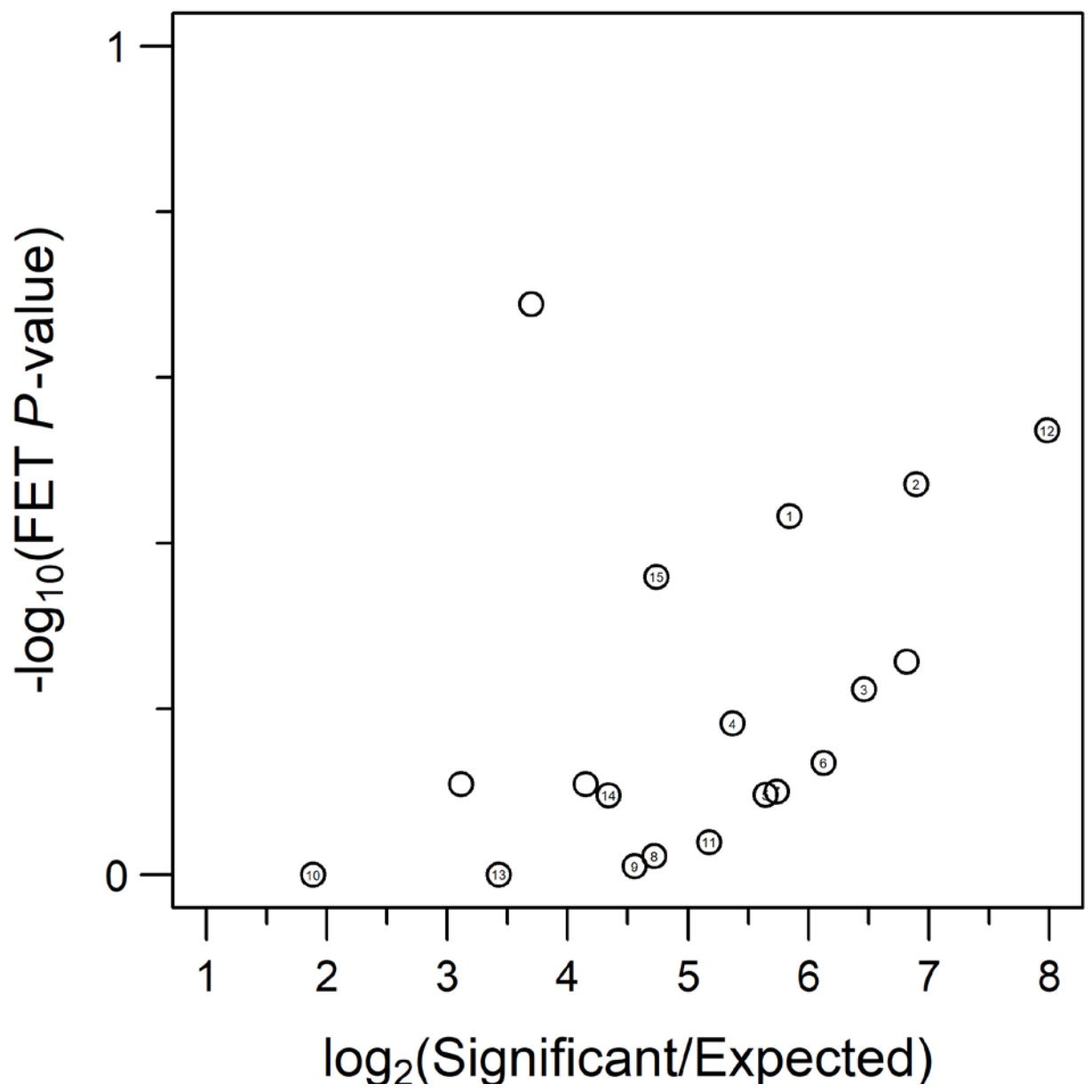

			Matches			P−values		
	Pathways	Size	Total	Significant	Expected	FET	EASE	Γ
1	Unsaturated Fatty Acids	265	125	15	0.26	0.37	0.47	0.11
2	Oxygenated hydrocarbons	17	11	2	0.02	0.34	0.71	0.14
3	Amino Fatty Acids	23	18	2	0.02	0.60	0.87	0.17
4	Dicarboxylic acids	49	20	2	0.05	0.66	0.90	0.18
5	C10 isoprenoids	81	49	4	0.08	0.80	0.91	0.19
6	Fatty aldehydes	29	23	2	0.03	0.73	0.93	0.20
7	Branched Fatty Acids	38	26	2	0.04	0.79	0.95	0.21
8	Short-chain acyl carnitines	77	41	2	0.08	0.95	0.99	0.28
9	Oxo Fatty Acids	43	32	1	0.04	0.98	1.0	1.0
10	Hydroxy Fatty Acids	274	112	1	0.27	1.0	1.0	1.0
11	Phenylpropanoic acids	28	21	1	0.03	0.91	1.0	1.0
12	Cinnamic acids	4	3	1	0.00	0.29	1.0	1.0
13	C15 isoprenoids	94	58	1	0.09	1.0	1.0	1.0
14	Fatty alcohols	50	14	1	0.05	0.80	1.0	1.0
15	N-acyl ethanolamines	38	5	1	0.04	0.44	1.0	1.0

Figure S6 Pathway enrichment analysis of features from Cluster #7 using the RefMet database. Pathway enrichment was evaluated using Fisher's exact test (FET). The significant set comprised 134 isotopologue D/H pairs assigned to Cluster #7, selected from a total of 2,691 pairs; the remaining pairs were used as the background set. The left panel shows a scatter plot of enriched pathways, with the shaded region indicating significantly enriched pathways ($p < 0.05$). The right panel lists the top 15 enriched pathways ranked by statistical significance.

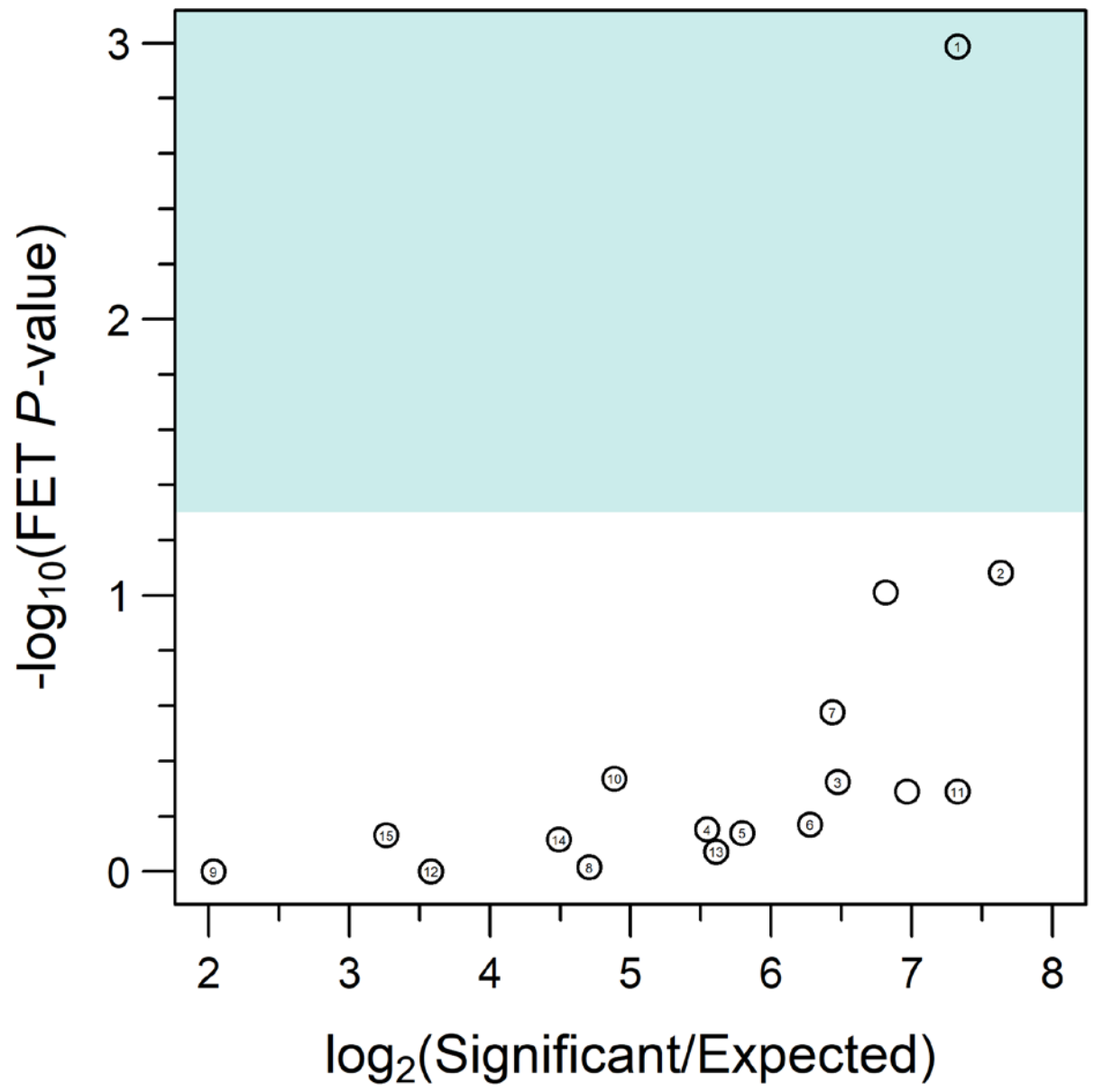

	Pathways	Size	Matches			P-values		
			Total	Significant	Expected	FET	EASE	Γ
1	Short-chain acyl carnitines	77	41	11	0.07	0.0010	0.0035	0.11
2	Oxygenated hydrocarbons	17	11	3	0.02	0.083	0.29	0.12
3	Branched Fatty Acids	38	26	3	0.03	0.47	0.73	0.16
4	Unsaturated Fatty Acids	265	125	11	0.24	0.70	0.80	0.17
5	C10 isoprenoids	81	49	4	0.07	0.72	0.87	0.19
6	Fatty aldehydes	29	23	2	0.03	0.68	0.91	0.21
7	Monoalkylamines	13	3	1	0.01	0.27	1.0	1.0
8	Oxo Fatty Acids	43	32	1	0.04	0.97	1.0	1.0
9	Hydroxy Fatty Acids	274	112	1	0.24	1.0	1.0	1.0
10	C18 steroids	38	6	1	0.03	0.46	1.0	1.0
11	Fatty lactones	7	7	1	0.01	0.51	1.0	1.0
12	C15 isoprenoids	94	58	1	0.08	1.0	1.0	1.0
13	Amino Fatty Acids	23	18	1	0.02	0.85	1.0	1.0
14	Fatty alcohols	50	14	1	0.04	0.77	1.0	1.0
15	Wax monoesters	117	13	1	0.10	0.74	1.0	1.0

Figure S7 Pathway enrichment analysis of features from Cluster #10 using the RefMet database. Pathway enrichment was evaluated using Fisher's exact test (FET). The significant set comprised 76 isotopologue D/H pairs assigned to Cluster #10, selected from a total of 2,691 pairs; the remaining pairs were used as the background set. The left panel shows a scatter plot of enriched pathways, with the shaded region indicating significantly enriched pathways ($p < 0.05$). The right panel lists the top 15 enriched pathways ranked by statistical significance.

Table S2 Theoretical *m/z* values of monoisotopic and single-^{13}C $[M+H]^+$ peaks and expected $^{13}C/^{12}C$ intensity ratios for the eight putative 19D-labeled acylcarnitines

Elemental formula	Theo. *m/z* ($[M+H]^+$, ^{12}C)	Theo. *m/z* ($[M+H]^+$, single-^{13}C)	No. of C atoms	Theo. $^{13}C/^{12}C$ (%)
$C_{10}D_{19}O_5N$	253.25286	254.25621	10	10.82
$C_{11}H_2D_{19}O_5N$	267.26851	268.27186	11	11.90
$C_{12}H_4D_{19}O_5N$	281.28416	282.28751	12	12.98
$C_{11}D_{19}O_5N$	265.25286	266.25621	11	11.90
$C_{12}H_2D_{19}O_5N$	279.26851	280.27186	12	12.98
$C_{13}H_4D_{19}O_5N$	293.28416	294.28751	13	14.06
$C_{11}D_{19}O_6N$	281.24777	282.25113	11	11.90
$C_{12}D_{19}O_6N$	293.24777	294.25113	12	12.98

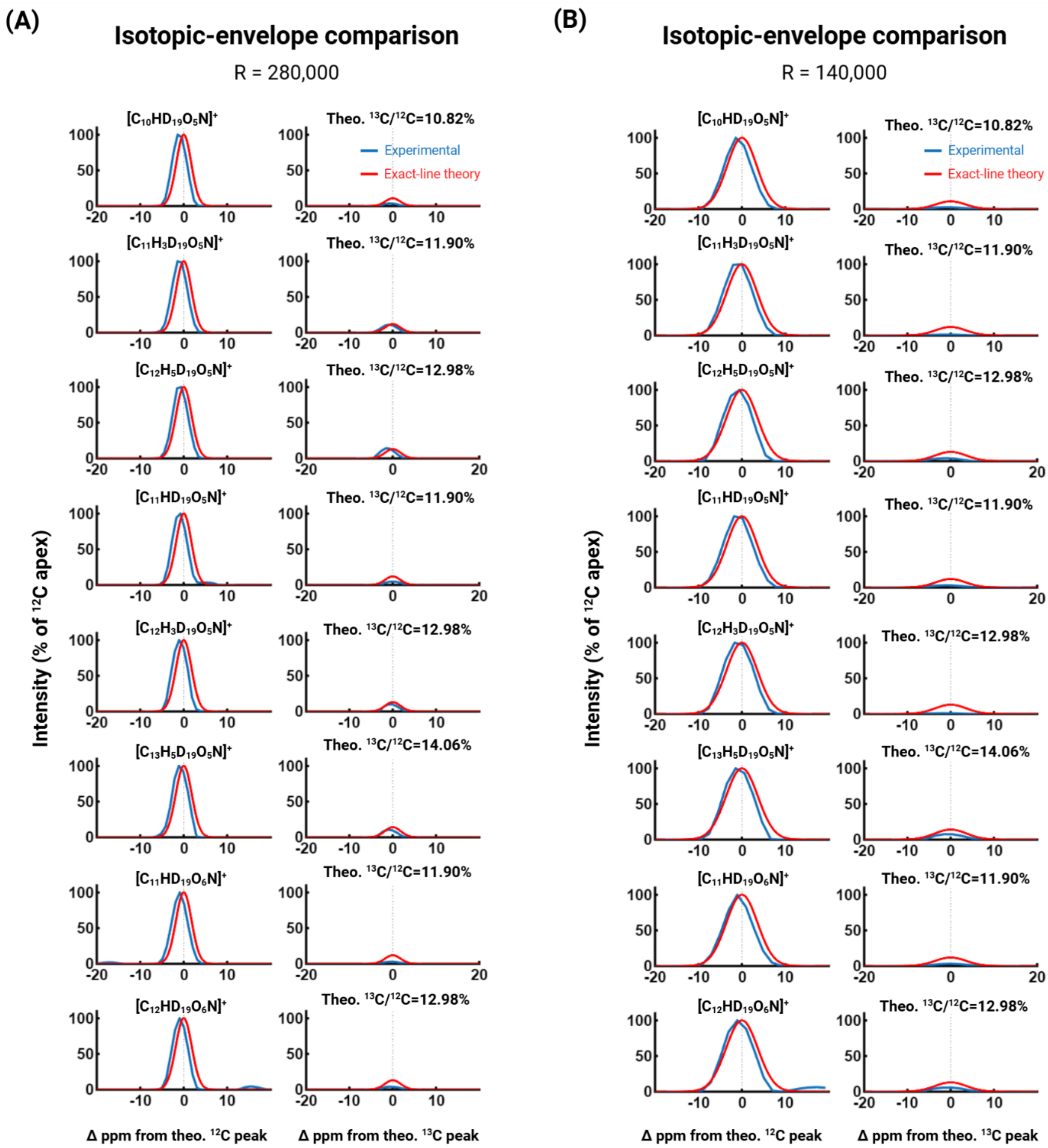

Figure S8 Isotopic-envelope comparisons for eight putative 19D-labeled acylcarnitines. (A) Representative breath samples from the Swiss discovery cohort were compared with theoretical profiles generated at a resolution of 280,000. (B) Representative breath samples from the Chinese validation cohort were compared with theoretical profiles generated at a resolution of 140,000. In both panels, comparisons are shown for the ^{12}C and corresponding ^{13}C windows, with intensities normalized to the ^{12}C apex. The eight putative 19D-labeled acylcarnitines and their theoretical exact-line isotope parameters are listed in **Table S2**.

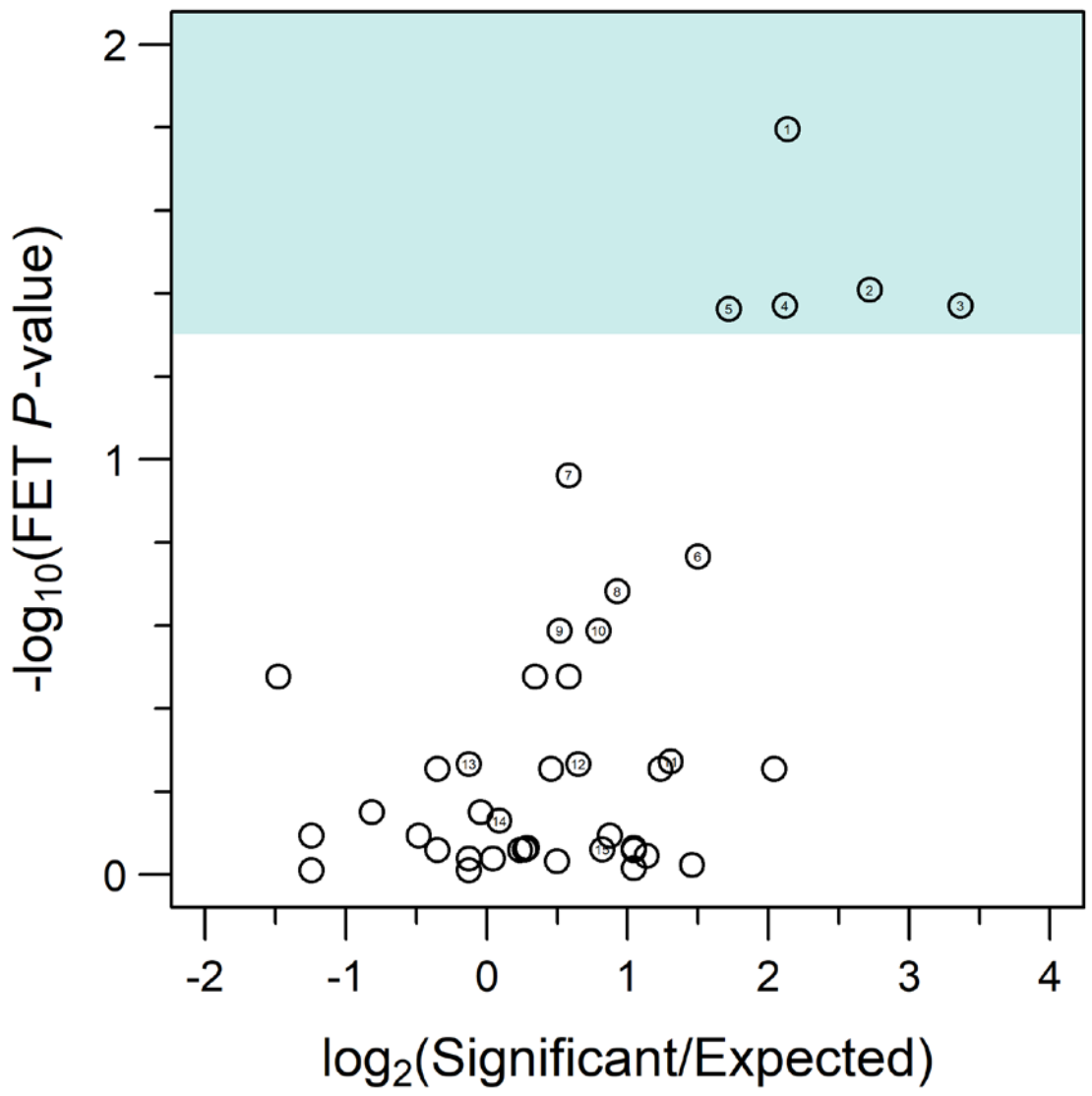

	Pathways	Size	Matches			P−values		
			Total	Significant	Expected	FET	EASE	Γ
1	Glycine, serine and threonine metabolism	30	12	8	1.82	0.016	0.056	0.022
2	Butanoate metabolism	15	9	6	0.91	0.039	0.13	0.025
3	Valine, leucine and isoleucine biosynthesis	8	7	5	0.49	0.043	0.16	0.026
4	Propanoate metabolism	19	7	5	1.15	0.043	0.16	0.026
5	Pyruvate metabolism	20	5	4	1.21	0.043	0.20	0.027
6	Valine, leucine and isoleucine degradation	35	12	6	2.12	0.17	0.35	0.035
7	Cysteine and methionine metabolism	33	4	3	2.00	0.11	0.40	0.037
8	One carbon pool by folate	26	5	3	1.58	0.21	0.53	0.047
9	Glycolysis or Gluconeogenesis	23	3	2	1.40	0.26	0.70	0.067
10	Glutathione metabolism	19	3	2	1.15	0.26	0.70	0.067
11	Fructose and mannose metabolism	20	8	3	1.21	0.53	0.80	0.087
12	beta-Alanine metabolism	21	5	2	1.27	0.54	0.87	0.11
13	Biosynthesis of unsaturated fatty acids	36	5	2	2.19	0.54	0.87	0.11
14	Glyoxylate and dicarboxylate metabolism	31	7	2	1.88	0.74	0.94	0.15
15	Alanine, aspartate and glutamate metabolism	28	13	3	1.70	0.87	0.96	0.17

Figure S9 Pathway enrichment analysis of features from Clusters #4, #7, and #10 using the RefMet database. Pathway enrichment was evaluated using Fisher's exact test (FET). The significant set comprised 315 isotopologue D/H pairs assigned to Clusters #4, #7, and #10, selected from a total of 2,691 pairs; the remaining pairs were used as the background set. The left panel shows a scatter plot of enriched pathways, with the shaded region indicating significantly enriched pathways ($p < 0.05$). The right panel lists the top 15 enriched pathways ranked by statistical significance.

Section 2: Experimental Section

Participants

The first cohort for discovery study was recruited in Switzerland. The discovery study was approved by the Ethics Committee of North-Western and Central Switzerland (ID: 2020-00778) and was registered in International Clinical Trial (https://clinicaltrials.gov/, NCT05274334). To further assess the reproducibility and broader applicability of this approach, we repeated the study in China with an independent validation cohort. The validation study was approved by the Institutional Review Board of Jinan University (ID: JNUKY-2023-0059). Participants who reported regular medication use or pregnancy were excluded. Each participant gave written consent before participating in the study. This study was conducted in accordance with Good Clinical Practice and the Declaration of Helsinki, and no unexpected or unusually high safety risks were encountered.

Study Design

All participants were instructed to fast for at least eight hours before breath sampling to minimize the influence of preceding food intake on baseline measurements. Upon arrival at the breath test laboratory, participants rinsed their mouths three times with clean tap water, followed by four baseline measurements. Participants then ingested deuterium oxide (D_2O, 99.9 atom % D, Sigma-Aldrich, Germany). The administered D_2O dose corresponded to 70% of 2 g/kg total body water, with total body water approximated as 50% of body weight. For dose calculation, total body water was approximated, although actual body water fraction may vary between individuals depending on factors such as sex, age, and adiposity. Two hours after D_2O ingestion, participants were provided with 300 g of yogurt containing approximately 10% carbohydrates, 3% protein, and 10% fat, predominantly saturated. The study design is shown in **Figure S10**. Each participant was scheduled to provide 20 breath measurements during this study: 1) four baseline breath measurements were performed after 8 hours of fasting; 2) following the ingestion of deuterated water, breath samples were collected every 15 minutes for a total of eight measurements over the subsequent two hours; 3) the remaining eight exhalations were taken over the next 2 hours after ingested yogurt.

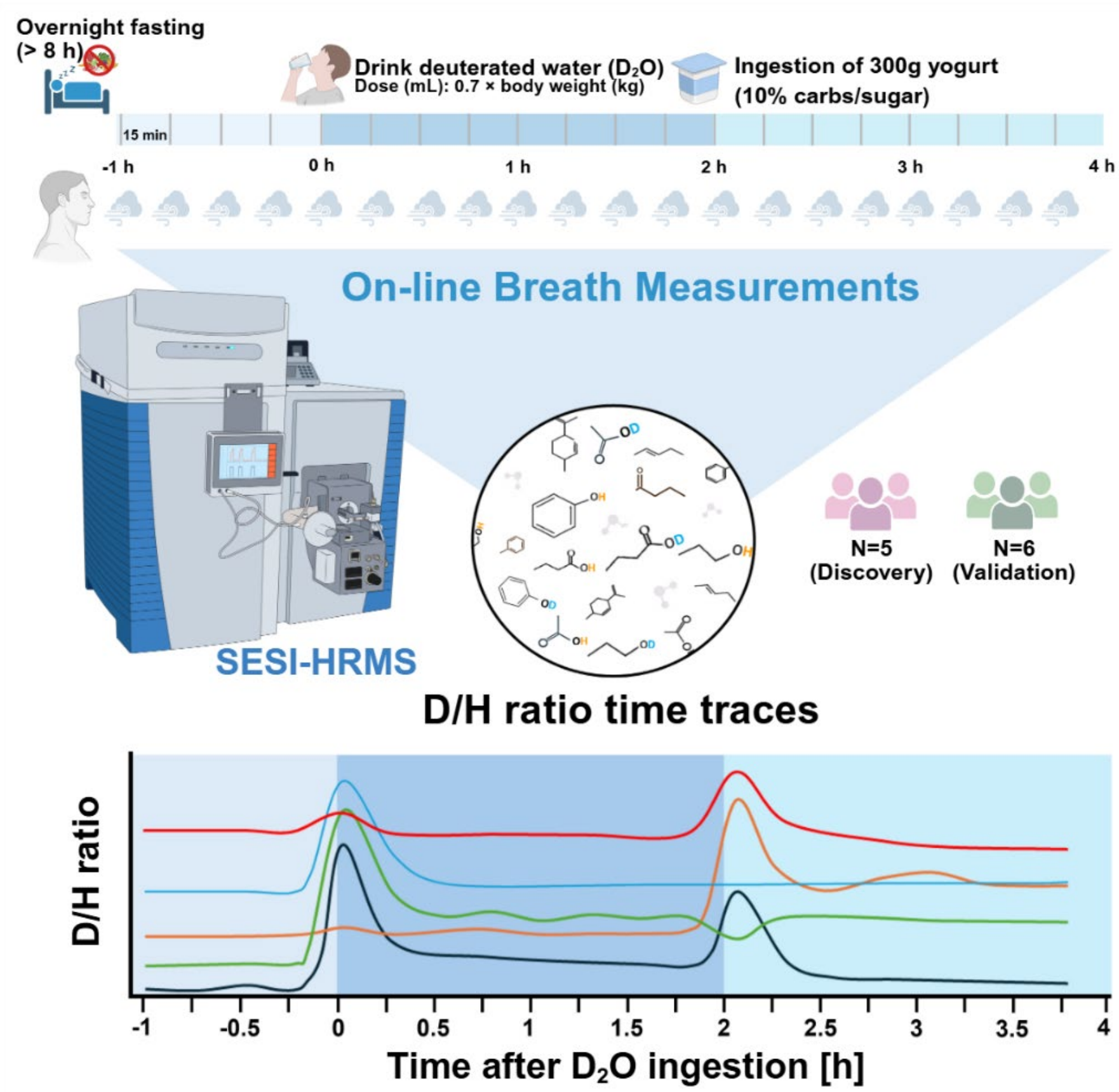

Figure S10 Schematic diagram of study design. After an overnight fast (>8 h), all participants provided one breath sample at 15 min intervals from -1 to 4 h, for a total of 20 breath data points. They ingested deuterated water at t = 0 h and consumed yogurt at t = 2 h. SESI-HRMS enabled real-time detection of a wide range of volatile and semivolatile metabolites in exhaled breath, including D-labeled isotopologues. The representative D/H ratio time-resolved signal traces illustrate the dynamic change of metabolites *in vivo* after the intake of D_2O and yogurt. Shaded regions with different color highlight the post-intake periods.

On-line Breath Measurement

The on-line breath measurements were performed following the standardized operational procedures.[1] The breath interface (Exhalion, Fossiliontech, Spain) was integrated into the analytical platform to assist in the control and reproducibility of the exhalation maneuver. During sampling, it provided real-time visual feedback and continuously recorded CO_2 (%), pressure drop (mbar), exhalation flow rate (L/min), and exhaled volume (L). Before formal breath measurements, each participant performed a rehearsal of the standardized breathing maneuver under researcher guidance. The participants exhaled at a constant flow rate of 7 L/min and stopped before breathing becomes exhausting or uncomfortable. A complete breath measurement requires at least 12 such exhalations (6 each in positive and negative

mode), and typically takes ~10 min. The commercially disposable plastic mouthpiece (MicroGard™, Vyaire Medical, Product no.: 892103, Germany) was used in this study.

Instrument Setting and Quality Control

The Swiss and Chinese cohorts were analyzed using the standardized real-time breath analysis platform established in our previous work for multicenter studies.[2] A harmonized analytical workflow and broadly comparable acquisition settings were used at both study sites. The platform consisted of three main components: (1) an expiratory interface (Exhalion, Fossiliontech, Spain), (2) an atmospheric-pressure secondary electrospray ionization source (Super SESI, Fossiliontech, Spain), and (3) a high-resolution mass spectrometer (Thermo Fisher Scientific, USA). For the Swiss discovery cohort, a Q Exactive Plus with an enhanced resolution of 280,000 was used, and for the Chinese validation cohort, a Q Exactive with a normal resolution of 140,000 was used. The breath sample was passed through a sample line heated to 130 °C into the ionization chamber of the SESI source to minimize compound adsorption onto the tubing walls. The ionized analytes were then directly analyzed by high-resolution mass spectrometry. Mass spectrometric acquisition was performed in full-scan mode over a mass range of 70–1000, with a spray voltage of ± 2.8 kV, sheath gas flow of 60, auxiliary gas flow of 2, capillary temperature of 275 °C, and 2 microscans. During the study period, the same quality control procedure was applied at both sites: in positive mode, 100 ppb α-pinene standard gas was measured through the SESI-HRMS platform before the first daily breath measurement. In addition, weekly external mass calibration was performed using the commercial calibration solution (Pierce™ ESI Positive/Negative, Thermo Fisher Scientific, USA).

Data Analysis and Statistics

Pre-processing Mass spectrometric RAW files were preprocessed using in-house C# console applications based on Thermo Fisher Scientific's RawFileReader (version 5.0.0.38) and MATLAB (R2022a–2025b; MathWorks Inc., USA). For each breath measurement, a representative mass spectrum was generated by averaging all scans acquired during exhalation windows with CO_2 concentrations >3% in order to reduce the contribution of early exhaled dead-space air while retaining a broader range of breath metabolites.[1, 3] Apodization was applied to find and remove satellite peaks, and the spectra were recalibrated using a polynomial fitting. The centroided data were then

refined using the KS-density function to achieve a mass accuracy of ±1 ppm. Candidate molecular formulae were assigned to detected features based on accurate mass, considering only C, H, N, and O atoms, and the corresponding deuterated formulae were subsequently derived from the expected mass shifts of D and H. This workflow yielded a data matrix comprising 213 samples, with 4,926 features detected in positive ion mode and 1,379 features detected in negative ion mode.

Post-processing Deuterium incorporation was first screened by searching for D/H isotopologue pairs differing by $\Delta m = n \times 1.0063$ Da (±0.0005 Da), corresponding to replacement of n hydrogen atoms by deuterium (n = 1, 2, 3, …). For each sample and time point, the D/H ratio was calculated as the signal-intensity ratio of the D-labeled feature to its corresponding unlabeled feature. To identify groups of isotopologue pairs with similar temporal behavior across the baseline, post-D_2O, and postprandial phases, we applied a noise-augmented von Mises–Fisher mixture model (NAvMix; R package navmix v0.2.1) following the methodological framework of Maik et al.[4, 5] Each resulting cluster therefore represents a group of D/H ratio time traces sharing a similar temporal shape over the course of the study. NAvMix was first run with a maximum of 20 clusters, and model fit was evaluated across 1–20 cluster solutions using the Bayesian Information Criterion (BIC). Because the BIC profile reached a clear plateau after approximately ten clusters (**Figure S2**), NAvMix was then rerun with K fixed at 10 to obtain final assignments for each isotopologue pair. For downstream interpretation, we prioritized three representative positive-response clusters (Clusters #4, #7, and #10; 315 isotopologue pairs in total), corresponding to a sustained early post-D_2O response, a sustained postprandial increase, and a transient postprandial surge, respectively. Where relevant, enrichment analyses were performed using Fisher's exact test, with the selected cluster-assigned features used as the significant set and the remaining D/H pairs used as background.

Putative Metabolite Annotation Strategy

Potential compound classes were first explored using the "lipids and non-lipids main chemical class" metabolite sets in MetaboAnalystR (version 3.0.3; https://www.metaboanalyst.ca). Candidate annotations were then cross-referenced against HMDB (https://www.hmdb.ca), Pubchem (https://pubchem.ncbi.nlm.nih.gov/), KEGG (https://www.genome.jp/kegg/kegg1.html) and OMIM (https://www.omim.org/) database using "CrossReferencing" function of MetaboAnalystR. Final putative

annotations were established using a stepwise prioritization framework that considered: (i) consistency with the study design and the observed temporal behavior, including the fasting/feeding intervention, the D/H ratio trajectory, the cluster-level pattern, and the known biochemical context of the candidate metabolite; and (ii) concordance with prior evidence from the literature, MS/MS data, or authentic standards, where available. Features retained after this process were reported as putative annotations and were used only for cautious, evidence-informed, and hypothesis-generating biological interpretation. For the highly labeled acylcarnitines, this assignment was further supported by the targeted exact-line isotope validation described below.

Targeted Exact-line Isotope Validation

Targeted isotope validation of highly labeled features was performed using a custom exact-line model implemented in MATLAB R2025b (MathWorks Inc., USA). This analysis focused specifically on the eight putative 19D-labeled acylcarnitines summarized in **Table S2.** Elemental formulas were used to calculate the theoretical *m/z* values of the monoisotopic $[M+H]^+$ peak and the corresponding single-^{13}C isotopic peak, as well as the expected $^{13}C/^{12}C$ intensity ratio. Theoretical ^{12}C and ^{13}C peak shapes were simulated as Gaussian profiles at Orbitrap resolving powers of 280,000 and 140,000, corresponding to the two acquisition settings used in this study. Only breath samples in which both the ^{12}C and corresponding ^{13}C centroids were detected within ±5 ppm of the theoretical *m/z* values were considered evaluable. Raw profile windows centered on the theoretical ^{12}C and ^{13}C positions (±10 ppm) were compared directly with the theoretical profiles without baseline correction, normalization, or ppm-shift optimization. The ^{12}C window, the ^{13}C window, and the combined ^{12}C + ^{13}C window were quantified using Pearson correlation, cosine similarity, normalized root-mean-square error (NRMSE), coefficient of determination (R^2), centroid ppm error, and profile-apex ppm error.

References

(1) Singh, K. D.; Tancev, G.; Decrue, F.; Usemann, J.; Appenzeller, R.; Barreiro, P.; Jaumà, G.; Macia Santiago, M.; Vidal de Miguel, G.; Frey, U.; Sinues, P. Standardization procedures for real-time breath analysis by secondary electrospray ionization high-resolution mass spectrometry. *Anal. Bioanal. Chem.* **2019**, *411* (19), 4883-4898.

(2) Gisler, A.; Singh, K. D.; Zeng, J.; Osswald, M.; Awchi, M.; Decrue, F.; Schmidt, F.; Sievi, N.; Chen, X.; Usemann, J.; et al. An Interoperability Framework for Multicentric Breath Metabolomic Studies. *iScience* **2022**, *25* (12), 105557.

(3) Suarez-Sipmann, F.; Bohm, S. H.; Tusman, G. Volumetric capnography: the time has come. *Curr Opin Crit Care* **2014**, *20* (3), 333-339.

(4) Pietzner, M.; Uluvar, B.; Kolnes, K. J.; Jeppesen, P. B.; Frivold, S. V.; Skattebo, Ø.; Johansen, E. I.; Skålhegg, B. S.; Wojtaszewski, J. F. P.; Kolnes, A. J.; et al. Systemic proteome adaptions to 7-day complete caloric restriction in humans. *Nat. Metab.* **2024**, *6* (4), 764-777.

(5) Grant, A. J.; Gill, D.; Kirk, P. D. W.; Burgess, S. Noise-augmented directional clustering of genetic association data identifies distinct mechanisms underlying obesity. *PLoS Genet.* **2022**, *18* (1), e1009975.
