# Supplementary material for: In Vivo Real-Time Metabolic Isotopic Tracing via Breath Analysis upon Deuterated Water Ingestion: A Proof-of-Concept Study
Source: ACS Cent Sci. 2026 Jun 25;12(8):1110–8. doi: 10.1021/acscentsci.6c00078 (PMC13523499; doi:10.1021/acscentsci.6c00078)
Supplement: Supplementary file 2 [file oc6c00078_si_004.pdf]

oc-2026-00078m.R1

Name: Peer Review Information for "In vivo real-time metabolic isotopic tracing via breath analysis upon deuterated water ingestion"

First Round of Reviewer Comments

Reviewer: 1

Comments to the Author

The manuscript "In vivo real-time metabolic isotopic tracing via breath analysis upon deuterated water ingestion" presents a new non-invasive method using high-resolution mass spectrometry (SESI-HRMS) to track metabolic activity in real time by detecting deuterium-labeled compounds in exhaled breath after participants drink deuterated water (D_2O). The study demonstrates that this approach can capture dynamic metabolic changes — including gut microbial fermentation, energy metabolism shifts, and glycemic responses. This is a well-written and scientifically interesting manuscript.

Here are the major points I am proposing for consideration:

- Sample size (N = 11): too small. The authors acknowledge this, but it would strengthen the paper to explicitly state the statistical power implications or at least acknowledge that the study is exploratory/hypothesis-generating in nature.
- Putative annotations: The reliance on MS1 accurate-mass data for metabolite annotation is a known limitation, and the authors acknowledge it. However, given that the entire biological interpretation (SCFAs, acylcarnitines, pyruvaldehyde) rests on these putative annotations, some other confirmation of the key annotated metabolites — even for a subset would be recommended (e.g. chemical standards).
- The "spike-then-drop" at t = 0 (lines 89–92): The interpretation that this reflects oral cavity D_2O clearance is reasonable, but presented as established fact. A brief acknowledgment that this is the most logic explanation (and that alternative interpretations exist) would be more rigorous.
- D_2O dosing: The dose is described as "2 g/kg body-water" with body water estimated as 0.5 × body weight (line 310). This is a rough estimate, and actual body water fraction varies significantly with gender, age, and adiposity. Given the biological interpretation depends

on D enrichment levels, acknowledging this approximation would strengthen the methods.

- Yogurt composition variability: The yogurt was "purchased from local market" (line 312) in two different countries. Differences in composition between Swiss and Chinese commercial yogurts (microbiota, carbohydrate content, fat profile) could introduce systematic confounds between cohorts. This should at minimum be acknowledged as a potential source of inter-cohort variability.
- Cluster assignment methodology: The NAvMix clustering approach is sophisticated but not widely known. A brief intuitive explanation of what the clusters represent (beyond the BIC criterion for K = 10) would help readers follow the logic. It is also worth clarifying why only Clusters #4, #7, and #10 were selected — the criterion of "increasing trend after D_2O or food ingestion" (line 362) needs to be defined more precisely or quantitatively.
- Figure 3B: The comparison between experimental and simulated spectra is visually convincing, but the figure only shows one representative subject. Showing agreement across more subjects or providing a quantitative goodness-of-fit metric would make this claim more robust.
- No breath blank or ambient air control is described. Since SESI-HRMS samples breath in real time, it would be important to confirm that detected D-labeled features are not artifacts from ambient deuterium-containing compounds or instrument background. Was any ambient air sampling performed?
- The CO_2 threshold for end-tidal selection (>3%, line 346) is on the lower end — many breath studies use >4–5% to ensure true end-tidal air. The rationale for the 3% cutoff should be justified, as it may affect the metabolite profiles captured.
- Inter-instrument variability: The two cohorts used SESI-HRMS platforms at two different sites (Switzerland and China). Were the instruments calibrated identically? Were cross-site quality control samples used? This is particularly relevant since D/H ratios are being compared across cohorts.
- The exhalation flow rate of 7 L/min (line 330) is relatively high and may not be sustainable for all participants equally, potentially introducing variability in breath dilution. How was compliance with this flow rate monitored and ensured?
- Fasting duration variability: Participants fasted "at least eight hours" — this means fasting duration varied between individuals, which could affect baseline metabolic state and D/H ratios. Was baseline fasting duration recorded and considered in the analysis?
- The relationship to deuterium metabolic imaging (DMI) is mentioned only via reference 3 (de Feyter et al., 2018). Given that DMI is an emerging and high-profile field, a sentence or two explicitly positioning SESI-HRMS breath analysis as a complementary or orthogonal approach to DMI could strengthen the introduction and broaden the paper's appeal.

- The gut microbiome angle is touched on throughout but never fully developed. Given that SCFAs and their D-labeling patterns are explicitly linked to microbial fermentation, and that inter-individual differences are attributed to microbial variability, the absence of any microbiome characterization of the participants is a notable gap — particularly for the validation cohort in China, where gut microbiome composition is known to differ substantially from European populations. This is worth acknowledging explicitly in the limitations.
- Clinical translation pathway is underdeveloped. A slightly more explicit discussion of what disease areas or clinical questions this technology could realistically address in the near term would strengthen the impact of the Conclusions section. The diabetes/oncogenesis link via pyruvaldehyde is a good start, but could be expanded slightly.

Minor Points:

- Table 1 is missing units for age — it lists numerical values but no "years" header, unlike weight (kg) and BMI (kg/m^2).
- Line 334: "The commercially disposable plastic mouthpiece...were used" — should be "was used."
- Line 72: The sentence "Yielding a total of 213 breath measurements" is a fragment — it should be connected to the preceding sentence with a comma or rewritten (e.g., "...except for one subject, who provided 13 measurements, yielding a total of 213 breath measurements.")
- Line 163: "included" should be "including" — "Other metabolites putatively annotated in this cluster likewise showed transient postprandial increases, *included* γ-aminobutyric acid..."
- The correspondence between Figure numbers in the main text and supplementary figures (S1–S9) is dense. A brief supplementary methods section explaining what each supplementary figure shows would improve navigability for readers.

Author's Response to Peer Review Comments:

Response to Reviewer

Journal: ACS Central Science

Manuscript ID: oc-2026-00078m

Title: "*In vivo* real-time metabolic isotopic tracing via breath analysis upon deuterated water ingestion"

Author(s): Yin, Zhihong; Singh, Kapil Dev; Tang, Zhifeng; Richard, Mélina; Zeng, Jiafa; Frey, Urs; Sinues, Pablo; Li, Xue

Dear Editor and Reviewer,

We sincerely thank you for the careful evaluation of our manuscript and for the constructive comments. We have carefully revised the manuscript accordingly. Below, we provide a point-by-point response and indicate the corresponding changes made in the revised manuscript.

To improve the organization and readability of the revised manuscript, the **Methods section** is now presented in the **Supplementary Information** rather than at the end of the main text. Accordingly, unless otherwise noted, all method-related revisions described below are incorporated in the **Supplementary Information Methods section**.

To facilitate your review of our responses and the revised manuscript, we have used the following color-coding approach in our response letter.

Blue text	Represents the original comments from the reviewer
Black text	Indicates our direct responses to the reviewer's comments
Red text	Shows the revised content that corresponds to the changes made in the revised manuscript

Reviewer's Comments:

The manuscript "*In vivo* real-time metabolic isotopic tracing via breath analysis upon deuterated water ingestion" presents a new non-invasive method using high-resolution mass spectrometry (SESI-HRMS) to track metabolic activity in real time by detecting

deuterium-labeled compounds in exhaled breath after participants drink deuterated water (D_2O). The study demonstrates that this approach can capture dynamic metabolic changes — including gut microbial fermentation, energy metabolism shifts, and glycemic responses. This is a well-written and scientifically interesting manuscript.

Here are the major points I am proposing for consideration:

Comment 1: *Sample size (N = 11): too small. The authors acknowledge this, but it would strengthen the paper to explicitly state the statistical power implications or at least acknowledge that the study is exploratory/hypothesis-generating in nature.*

Response: We are grateful for this constructive comment and agree with the reviewer's suggestion. Accordingly, we have implemented three key revisions in the manuscript:

(1) The **title** has been revised to (Page 1, line 1, in clean version of revised manuscript): "*In vivo* real-time metabolic isotopic tracing via breath analysis following deuterated water ingestion: a proof-of-concept study" to explicitly frame this work as a proof-ofconcept study.

(2) The limitations stated in the **Results and Discussion** section have been revised to (Pages 13-14, lines 327-331, in clean version of revised manuscript): "First, the present study should be regarded as exploratory and hypothesis-generating, given the modest sample size (N = 11) and restriction to healthy young volunteers. These factors constrain statistical power for broader biological inference and limit the generalizability of our findings to broader and clinically relevant populations." This revision clearly emphasizes the exploratory and hypothesis-generating nature of the work, as well as the need for larger and more diverse cohorts in future validation studies.

(3) The wording has been refined across the manuscript to better reflect its exploratory nature and avoid overinterpretation of the biological findings. For example, several more definitive expressions were replaced with more cautious wording, such as changing "highlights" to "suggests", "report on" to "may capture", "converged on" to

“appeared to converge on”, “are intrinsically more susceptible” to “may be more susceptible”, and “demonstrate” to “suggest”.

Comment 2: *Putative annotations: The reliance on MS1 accurate-mass data for metabolite annotation is a known limitation, and the authors acknowledge it. However, given that the entire biological interpretation (SCFAs, acylcarnitines, pyruvaldehyde) rests on these putative annotations, some other confirmation of the key annotated metabolites — even for a subset would be recommended (e.g. chemical standards).*

Response: We thank the reviewer for this important comment. We fully agree that the limitation of relying on MS1 accurate-mass data alone for putative annotation necessarily constrains the strength of the downstream biological interpretation.

At the same time, we would like to clarify that while direct confirmation using authentic standards or systematic MS/MS was not established in this study, the annotations were not assigned based exclusively on exact mass matching to databases. We implemented a stepwise prioritization framework to improve confidence in the putative annotations as much as possible within the available data. This framework included the following criteria: (1) consistency with the study design should be demonstrated, including the fasting/feeding intervention, the temporal trajectory of the D/H ratio, cluster-level behavior, and the known biological description of candidate metabolites; and (2) whether similar breath metabolites had previously been supported by MS/MS spectra, commercial standards, or relevant literature. We acknowledge that these criteria do not confirm metabolite identity definitively, but they do provide a more rigorous basis for the annotation and biological interpretation.

A representative example is the feature at *m/z* 87.04514 in negative ion mode, together with the corresponding feature at *m/z* 89.05971 in positive ion mode. Database matching yielded multiple possible candidate metabolites for these features (**Table 1**). We then used the above framework to prioritize the putative annotations.

Table 1 The multiple candidate metabolites for the features at *m/z* 87.04514 in negative mode and the feature *m/z* 89.05971 in positive mode

<table>
<tr><th>m/z</th><th>Adduct</th><th>Elemental formula</th><th>Putative annotations</th></tr>
<tr><td rowspan="4">87.04514</td><td rowspan="4">$[M-H]^-$</td><td rowspan="8">$C_4H_8O_2$</td><td>Butyric acid</td></tr>
<tr><td>Isobutyric acid</td></tr>
<tr><td>Acetoin</td></tr>
<tr><td>Methyl propionate</td></tr>
<tr><td rowspan="4">89.05971</td><td rowspan="4">$[M+H]^+$</td><td>Ethyl acetate</td></tr>
<tr><td>1-Hydroxy-2-butanone</td></tr>
<tr><td>Propyl formate</td></tr>
<tr><td>Isopropyl formate</td></tr>
</table>

As shown in **Figure 1**, the negative-ion feature at *m/z* 87.04514 and the positive-ion feature at *m/z* 89.05971 exhibited broadly similar temporal trajectories, including a sharp rise immediately after D_2O ingestion, followed by a gradual decrease over time and a subsequent increase observed after feeding. These concordant temporal patterns suggest that the two features reflect the same or closely related metabolite behavior. Within these candidates, this behavior is more consistent with a metabolite related to fatty-acid-associated metabolism and postprandial carbohydrate fermentation,[1, 2] such as butyric acid and isobutyric acid, than with rest alternative candidates.

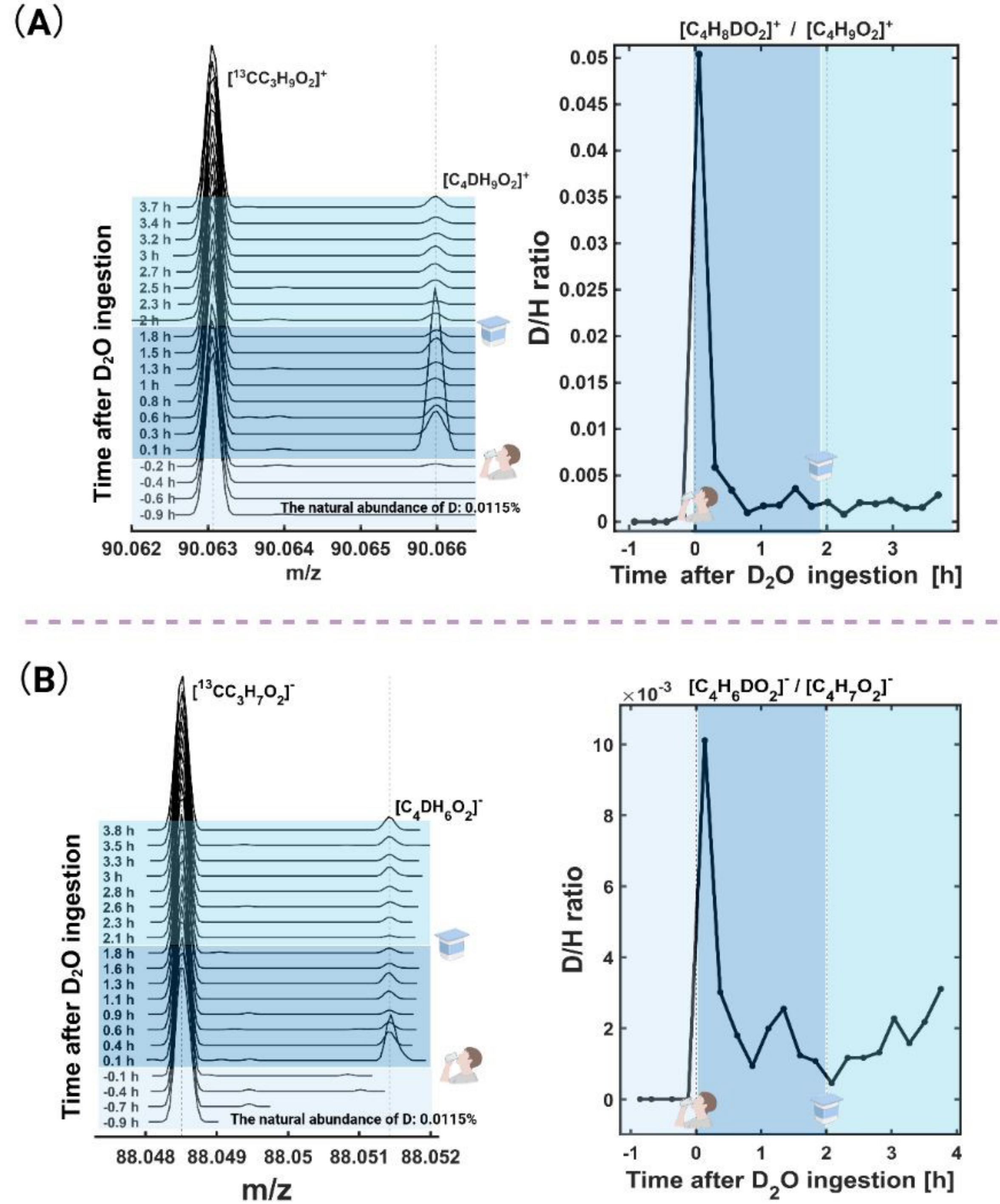

Figure 1. Time-resolved isotopic trajectories of the features putatively annotated as butyric acid in positive and negative ion modes after D_2O ingestion. (A) Feature at *m/z* 89.05971 ($[C_4H_9O_2]^+$). (B) Feature at *m/z* 87.04514 ($[C_4H_7O_2]^-$). Left panels show the high-resolution mass spectra from a representative subject, demonstrating isotopic resolution of ^{13}C and D-labeled peaks. The relative intensity normalized by the corresponding ^{13}C isotopic peak. Experimental peak positions align closely with the exact *m/z*

values (dashed lines). Right panels show the corresponding temporal profiles of the D/H ratio for the same participant. Dashed vertical lines indicate the time points of D_2O and yogurt ingestion.

To further inform this annotation, we additionally considered prior evidence from standard-based SESI-HRMS studies.[3-5] In our previous work, using the same SESIHRMS platform, we obtained standard- and MS/MS-based evidence (Figure 2, adapted from Ref. 3) supporting the identification for the positive-ion feature at *m/z* 89.0595 to butyric acid.[3] In addition, previous studies using SESI-HRMS of volatile short-chain fatty acid standards have reported a butyric acid feature at *m/z* 87.0453 ($[M-H]^-$) in negative ion mode[4] and butyric acid at *m/z* 89.0595 ($[M+H]^+$) in positive ion mode.[4]

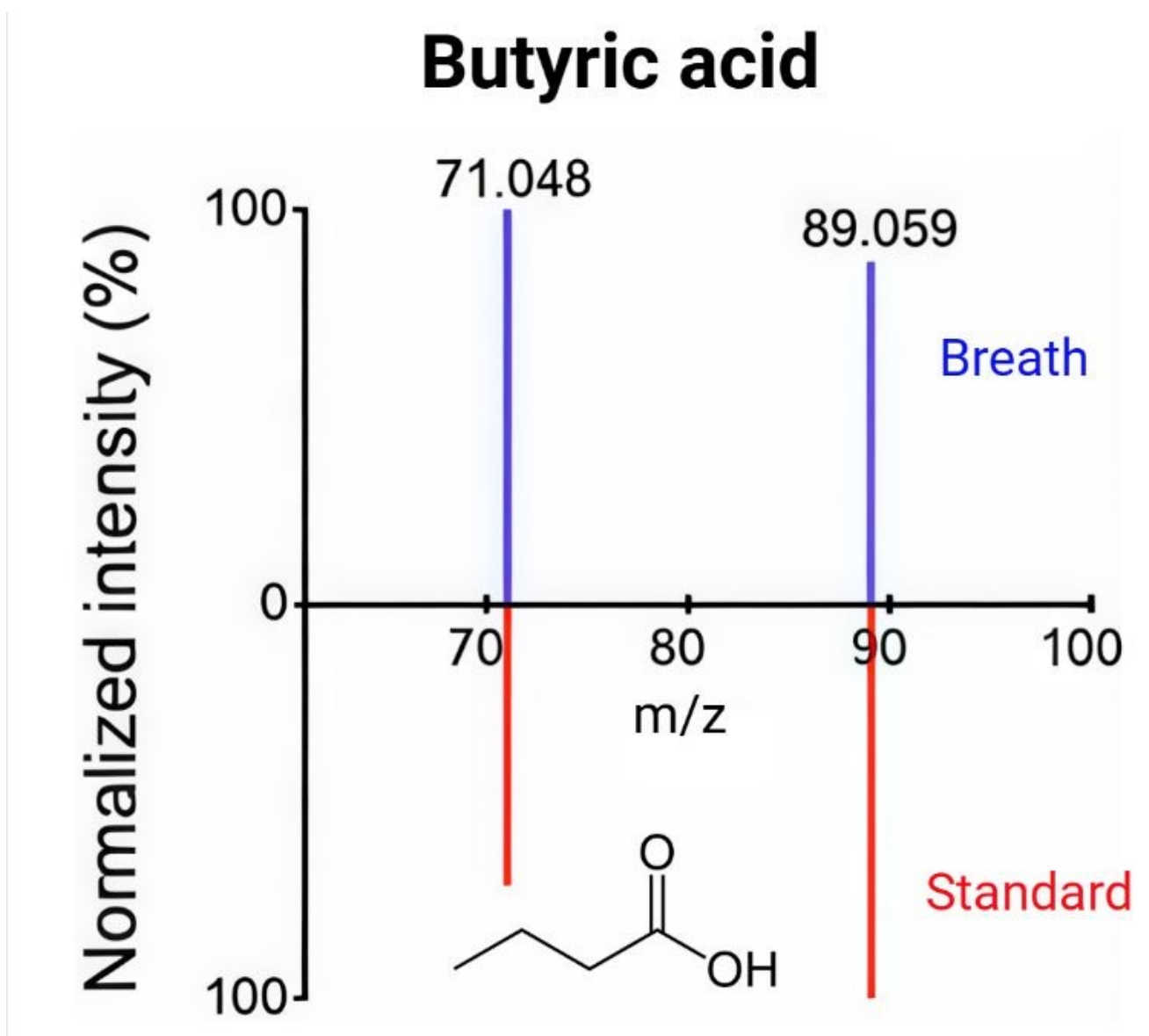

Figure 2 **I**dentification of breath metabolites. MS/MS spectra of butyric acid measured in breath (blue) and standards (red). Adapted from Zhifeng et al.[3]

Taken together, these converging lines of evidence support a butyric acid annotation as a biologically and analytically plausible candidate assignment for the negative-ion feature at *m/z* 87.04514 and the positive-ion feature at *m/z* 89.05971, while definitive discrimination among related candidates remains unresolved in the current dataset. We agree that these annotations should still be regarded as putative in the absence of direct confirmation with authentic standards in the current study. However, the available evidence indicates that the retained candidate annotations were cautiously prioritized.

In this sense, we believe that the related biological discussion can be retained as cautious, evidence-informed, and hypothesis-generating, while remaining explicitly provisional. Accordingly, we have revised the manuscript in four ways.

(1) In the **Results and Discussion** section, we revised the limitations to state (Page 14, lines 338-344, in clean version of revised manuscript): "Third, metabolite annotation in the current work remains putative because it relies primarily on accuratemass MS1 data, while interpretations involving potential microbial contributions are further limited by the absence of direct microbiome characterization. Future studies should combine multi-omics readouts (e.g., microbiome profiling) with systematic MS/MS validation, and confirmation with chemical standards for key metabolites to improve annotation confidence and mechanistic insight."

(2) The corresponding biological interpretations have been moderated throughout the text by consistently referring to these metabolites as putative annotations, avoiding definitive mechanistic language, and explicitly framing the related pathway-level discussion as tentative. Specifically, statements linking these features to SCFAs, acylcarnitines, and pyruvaldehyde-related metabolism were revised to use more cautious wording such as "consistent with", "suggestive of" or "potentially related to".

(3) Accordingly, a new subsection entitled **"Putative metabolite annotation strategy"** was added in the **Methods** section of the **Supplementary Information** (Pages S15S16, lines 252-268, in clean version of revised Supplemental information): "Potential compound classes were first explored using the "lipids and non-lipids main chemical class" metabolite sets in MetaboAnalystR (version 3.0.3; https://www.metaboanalyst.ca). Candidate annotations were then cross-referenced against HMDB (https://www.hmdb.ca), Pubchem (https://pubchem.ncbi.nlm.nih.gov/), KEGG (https://www.genome.jp/kegg/kegg1.html) and OMIM (https://www.omim.org/) database using "CrossReferencing" function of MetaboAnalystR. Final putative annotations were established using a stepwise prioritization framework that considered: (i) consistency with the study design and the observed temporal behavior, including the fasting/feeding intervention, the D/H ratio trajectory, the cluster-level pattern, and the

known biochemical context of the candidate metabolite; and (ii) concordance with prior evidence from the literature, MS/MS data, or authentic standards, where available. Features retained after this process were reported as putative annotations and were used only for cautious, evidence-informed, and hypothesis-generating biological interpretation. For the highly labeled acylcarnitines, this assignment was further supported by the targeted exact-line isotope validation described below."

Refences:

(1) Houten, S. M.; Violante, S.; Ventura, F. V.; Wanders, R. J. A. The Biochemistry and Physiology of Mitochondrial Fatty Acid β-Oxidation and Its Genetic Disorders. *Annu. Rev. Physiol.* **2016**, *78*, 23-44.

(2) Lupton, J. R. Microbial Degradation Products Influence Colon Cancer Risk: the Butyrate Controversy. *J. Nutr.* **2004**, *134* (2), 479-482.

(3) Tang, Z.; Yang, J.; Su, B.; Xu, X.; Luo, X.; Wang, H.; Zhang, K.; Huan, T.; Sinues, P.; Fang, M.; Li, X. Metabolite Fusion between Breath and Blood Enables More In-Depth Understanding of the Endogenous Metabolome. *Anal. Chem.* **2025**, *97* (36), 19427-19436.

(4) Lee, J. H. J.; Zhu, J. Optimizing Secondary Electrospray Ionization High-Resolution Mass Spectrometry (SESI-HRMS) for the Analysis of Volatile Fatty Acids from Gut Microbiome. *Metabolites* **2020**, *10* (9).

(5) Wuthrich, C.; Fan, Z.; Vergeres, G.; Wahl, F.; Zenobi, R.; Giannoukos, S. Analysis of volatile shortchain fatty acids in the gas phase using secondary electrospray ionization coupled to mass spectrometry. *Anal. Methods* **2023**, *15* (5), 553-561.

Comment 3: *The "spike-then-drop" at t = 0 (lines 89–92): The interpretation that this reflects oral cavity D_2O clearance is reasonable, but presented as established fact. A brief acknowledgment that this is the most logic explanation (and that alternative interpretations exist) would be more rigorous.*

Response: We appreciate the reviewer's insightful suggestion and agree with this comment. In the **Results and Discussion** section, the original sentence: "Immediately after ingestion, the ratio rose sharply to its maximum and then dropped by nearly 70% at the second data point (~15 min later). We attributed this rapid decrease to the clearance of residual D_2O in the oral cavity." has been revised to (Page 4, lines 109113, in clean version of revised manuscript): "Immediately after D_2O ingestion, the ratio peaked within 5 min and subsequently declined by nearly 70% at 15 min. Given that complete absorption of orally ingested D_2O and its equilibration with body fluids generally require approximately 75–120 min, we reasoned that this rapid decrease most likely reflects clearance of residual D_2O in the oral cavity.[4, 16]"

Relevant references have also been provided in the revised manuscript:

(4) Peronnet, F.; Mignault, D.; du Souich, P.; Vergne, S.; Le Bellego, L.; Jimenez, L.; Rabasa-Lhoret, R. Pharmacokinetic analysis of absorption, distribution and disappearance of ingested water labeled with D2O in humans. *Eur. J. Appl. Physiol.* **2012**, *112* (6), 2213-2222.

(16) Simon, D.; Patrik, S.; David, S. Rapid measurement of deuterium content of breath following oral ingestion to determine body water. *Physiol. Meas.* **2001**, *22* (4), 651.

Comment 4: *D_2O dosing: The dose is described as "2 g/kg body-water" with body water estimated as 0.5 × body weight (line 310). This is a rough estimate, and actual body water fraction varies significantly with gender, age, and adiposity. Given the biological interpretation depends on D enrichment levels, acknowledging this approximation would strengthen the methods.*

Response: We really appreciate your valuable suggestion. This point is now acknowledged in the **Methods** section of the Supplementary Information, which has been revised as follows (Page S12, lines 162-167, in clean version of revised Supplemental information): "Participants then ingested deuterium oxide (D_2O, 99.9 atom % D, Sigma-Aldrich, Germany). The administered D_2O dose corresponded to 70% of 2 g/kg total body water, with total body water approximated as 50% of body weight. For dose calculation,

total body water was approximated, although actual body water fraction may vary between individuals depending on factors such as sex, age, and adiposity."

Comment 5: *Yogurt composition variability: The yogurt was "purchased from local market" (line 312) in two different countries. Differences in composition between Swiss and Chinese commercial yogurts (microbiota, carbohydrate content, fat profile) could introduce systematic confounds between cohorts. This should at minimum be acknowledged as a potential source of inter-cohort variability.*

Response: We agree with the reviewer. In the present study, yogurt intake was standardized to 300 g, and the products showed broadly comparable macronutrient profiles (approximately 10% carbohydrates, 3% protein, and 10% fat, predominantly saturated). However, the yogurt used in the two cohorts was purchased locally. Therefore, compositional differences, such as differences in microbiota, may have persisted and contributed to inter-cohort variability.

We have now acknowledged this point in the revised **Results and Discussion** (Page 5, lines 121-130, in clean version of revised manuscript): "In addition, subtle differences were also observed across individuals in the magnitude and temporal profile of the later response. Given that short-chain fatty acids (SCFAs) originate primarily from gut microbial metabolism, [23, 24] these differences may reflect variation in gut microbial composition and metabolic activity among participants. Although yogurt intake was standardized to 300 g and the products showed broadly comparable macronutrient profiles (approximately 10% carbohydrates, 3% protein, and 10% fat, predominantly saturated), the yogurt used in the two cohorts was purchased locally. As a result, compositional differences, such as differences in microbiota, may have contributed to the variability observed between cohorts." Refences:

(23) Macfarlane, S.; Macfarlane, G. T. Regulation of short-chain fatty acid production. *Proc. Nutr. Soc.* **2003**, *62* (1), 67-72.

(24) Dalile, B.; Van Oudenhove, L.; Vervliet, B.; Verbeke, K. The role of short-chain fatty acids in microbiota-gut-brain communication. *Nat. Rev. Gastroenterol. Hepatol.* **2019**, *16* (8), 461-478.

Comment 6: *Cluster assignment methodology: The NAvMix clustering approach is sophisticated but not widely known. A brief intuitive explanation of what the clusters represent (beyond the BIC criterion for K = 10) would help readers follow the logic. It is also worth clarifying why only Clusters #4, #7, and #10 were selected — the criterion of "increasing trend after D2O or food ingestion" (line 362) needs to be defined more precisely or quantitatively.*

Response: We thank the reviewer for this helpful comment, and the manuscript has been revised accordingly as detailed below:

(1) In the **Methods** section of the Supplementary Information, the description of the NAvMix clustering procedure to read has been revised to (Page S15, lines 237-251, in clean version of revised Supplemental information): "To identify groups of isotopologue pairs with similar temporal behavior across the baseline, post-D_2O, and postprandial phases, we applied a noise-augmented von Mises-Fisher mixture model (NAvMix; R package navmix v0.2.1) following the methodological framework of Maik et al.[4, 5] Each resulting cluster therefore represents a group of D/H ratio trajectories sharing a similar temporal shape over the course of the study. The optimal number of clusters was guided by the Bayesian Information Criterion (BIC) profile, which showed a clear plateau after approximately ten clusters (**Figure S2**). Accordingly, K = 10 was selected as a compromise between model fit and interpretability. For downstream interpretation, we prioritized three representative positive-response clusters (Clusters #4, #7, and #10; 315 isotopologue pairs in total), corresponding to a sustained early post-D_2O response, a sustained postprandial increase, and a transient postprandial surge, respectively. Where relevant, enrichment analyses were performed using Fisher's exact test, with the selected cluster-assigned features used as the significant set and the remaining D/H pairs used as background." Refences:

(4) Macfarlane, S.; Macfarlane, G. T. Regulation of short-chain fatty acid production. *Proc. Nutr. Soc.* **2003**, *62* (1), 67-72.

(5) Grant, A. J.; Gill, D.; Kirk, P. D. W.; Burgess, S. Noise-augmented directional clustering of genetic association data identifies distinct mechanisms underlying obesity. *PLoS Genet.* **2022**, *18* (1), e1009975.

(2) In the **Results and Discussion** section of the revised manuscript, the relevant text has been modified (Pages 5-6, lines 137-149, in clean version of revised manuscript): "Noise-enhanced von Mises–Fisher mixture (NAvMix)[25] clustering partitioned these ratios into ten distinct temporal trajectory clusters (**Figures S2–S4**), each cluster representing a group of isotopologue pairs with similar response profiles across the experimental time course. Among these, we focused subsequent analyses on three representative clusters (Clusters #4, #7, and #10; 315 isotopologue pairs in total), which captured distinct and positive-response trajectories (**Figure 2A**): a sustained early positive response after D_2O administration (Cluster #4), a sustained positive postprandial increase (Cluster #7), and a sharp transient postprandial surge (Cluster #10). A summary of the dominant temporal characteristics of the positive-response clusters is provided in **Table S1**. The 315 isotopologue pairs assigned to these three representative clusters were subsequently mapped to the Human Metabolome Database (HMDB),[26] and the corresponding putative annotations are provided in **Supplementary Table 1**." Refences:

(25) Grant, A. J.; Gill, D.; Kirk, P. D. W.; Burgess, S. Noise-augmented directional clustering of genetic association data identifies distinct mechanisms underlying obesity. *PLoS Genet.* **2022**, *18* (1), e1009975.

(26) Wishart, D. S.; Guo, A.; Oler, E.; Wang, F.; Anjum, A.; Peters, H.; Dizon, R.; Sayeeda, Z.; Tian, S.; Lee, B. L.; et al. HMDB 5.0: the Human Metabolome Database for 2022. *Nucleic Acids Res.* **2022**, *50* (D1), D622-D631.

Accordingly, we added **Table S1** to the **Supplementary Information** (Page S7, lines 88-89, in clean version of revised Supplemental information):

Table S1 Summary of selected NAvMix clusters showing positive-response patterns after D_2O administration and/or yogurt ingestion

Cluster	n	Dominant temporal characteristic	Role in interpretation
1	80	immediate sharp post-D_2O spike followed by rapid return toward baseline	not prioritized; less representative of sustained labeling dynamics
4	105	sustained early positive response after D_2O administration	prioritized as a representative early D_2O-responsive pattern
5	170	very strong immediate post-D_2O spike followed by rapid return toward baseline	not prioritized; less representative of sustained labeling dynamics
7	134	sustained positive postprandial increase	prioritized as a representative sustained postprandial pattern
9	130	modest but sustained positive postprandial trend	considered but not prioritized, because the trajectory was less distinct
10	76	sharp transient postprandial surge followed by decline	prioritized as a representative transient postprandial pattern

Comment 7: *Figure 3B: The comparison between experimental and simulated spectra is visually convincing, but the figure only shows one representative subject. Showing agreement across more subjects or providing a quantitative goodness-of-fit metric would make this claim more robust.*

Response: We thank the reviewer for this valuable suggestion. We agree that showing only a single representative example in the original **Figure 3B** did not sufficiently demonstrate the robustness of the isotope-pattern agreement. The aim of this analysis was to support the assignment of highly deuterium-incorporated features by testing whether their observed isotope patterns were consistent with exact-line theoretical predictions.

To address this concern, we substantially revised this part of the manuscript. First, we replaced the original **Figure 3B** with a representative isotope-envelope comparison for one putative 19D-labeled acylcarnitine ($[C_{10}HD_{19}O_5N]^+$; *m/z*: 253.2529) from a representative breath sample, showing the agreement between the measured and simulated isotope patterns in both the ^{12}C and corresponding ^{13}C windows. Second, we added a new **Figure 3C** providing a cohort-level quantitative evaluation based on Pearson correlation analysis. This analysis was performed for the eight putative

19Dlabeled acylcarnitines across breath samples. Only samples in which both the ^{12}C and corresponding ^{13}C centroids were detected within ±5 ppm of the theoretical *m/z* values were considered evaluable. The resulting median r values were 0.956 for the ^{12}C window, 0.858 for the ^{13}C window, and 0.958 for the combined fit. Third, we replaced the original **Figure S8** with additional isotope-envelope comparisons for eight putative 19D-labeled acylcarnitines. Theoretical ^{12}C and ^{13}C peak shapes were simulated as Gaussian profiles at Orbitrap resolving powers of 280,000 and 140,000, corresponding to the acquisition settings used for the Swiss discovery cohort (Q Exactive Plus) and the Chinese validation cohort (Q Exactive), respectively. Accordingly, we have implemented four key revisions in the manuscript:

(1) In the **Results and Discussion** section, the original sentence: "The assignment of these 19D-labeled acylcarnitines was further supported by the excellent agreement between measured and simulated isotopic envelopes for both the ^{12}C and ^{13}C monoisotopic peaks (Figure 3B; Figure S8)." has been revised to (Pages 9-10, lines 249-255, in clean version of revised manuscript): "The assignment of these putative 19D-labeled acylcarnitines was supported by the close agreement between measured and simulated isotopic patterns in both the ^{12}C and corresponding ^{13}C windows (**Figure 3B; Figure S8**). This agreement was further supported by Pearson correlation analysis of the eight putative 19D-labeled acylcarnitines observed across breath samples, with median r values of 0.956 for the ^{12}C window, 0.858 for the ^{13}C window, and 0.958 for the combined fit (**Figure 3C**)."

(2) We revised the original **Figure 3B** to (Page 11, lines 268-281, in clean version of revised manuscript):

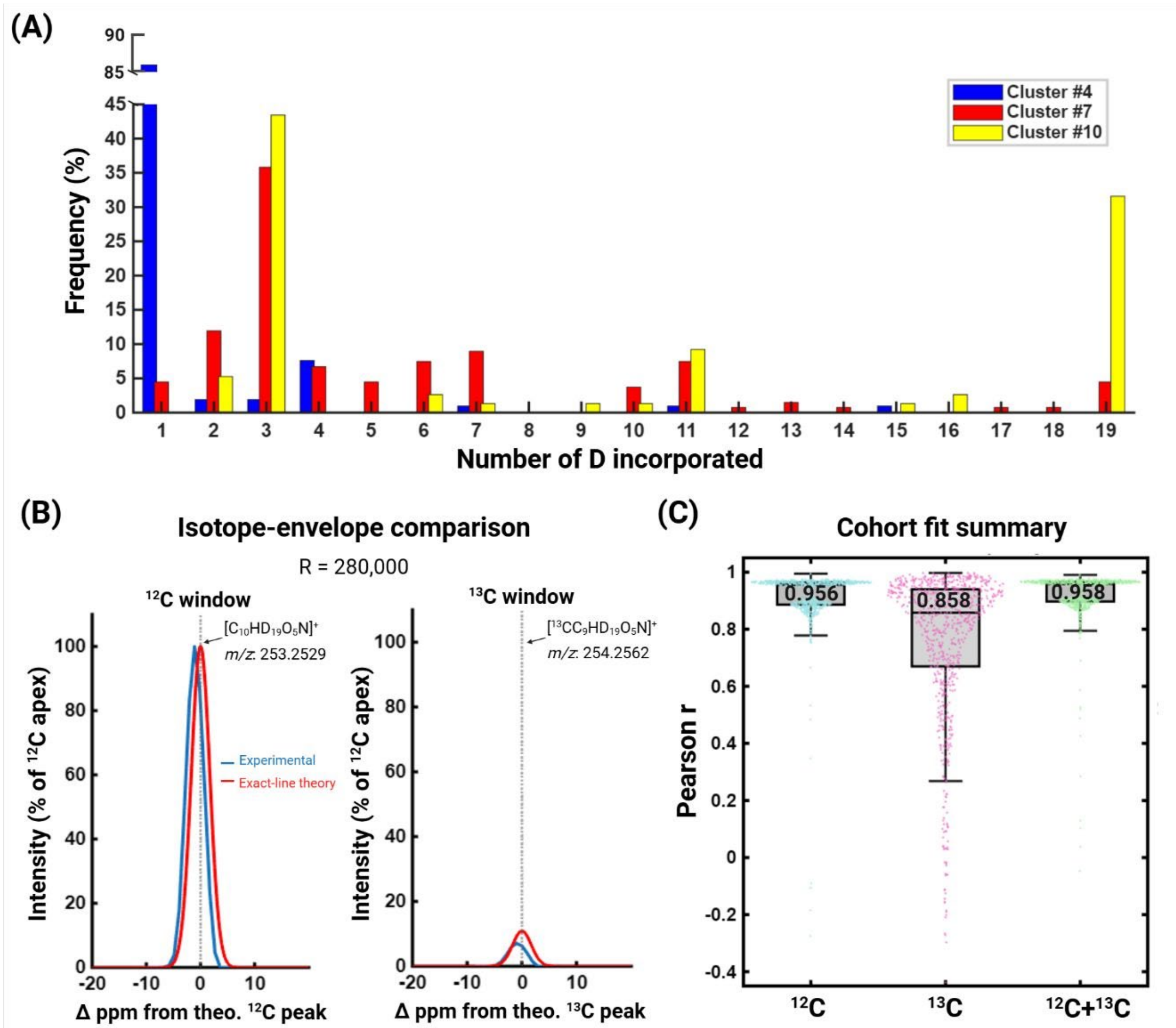

Figure 3 Deuterium incorporation patterns and isotope-pattern validation of high-level D-incorporation features. (A) Distribution of the number of incorporated deuterium atoms (n) across three representative time-course clusters (Clusters #4, #7, and #10). Histograms represent the fraction of features within each cluster that exhibit a given level of D incorporation. The y-axis is truncated (45–85%) to improve visualization of differences among lower-frequency incorporation levels. (B) Isotope-envelope comparison for one putative 19D-labeled acylcarnitine ($[C_{10}HD_{19}O_5N]^+$; *m/z*: 253.2529) in a representative breath sample. Experimental profiles are compared with exact-line theoretical profiles simulated at a resolution of 280,000 for the ^{12}C and corresponding ^{13}C windows, with intensities normalized to the ^{12}C apex. Additional putative 19D-labeled acylcarnitine comparisons are shown in **Figure S8**. (C) Pearson correlation analysis comparing measured and theoretical isotope patterns for putative 19D-labeled acylcarnitines across breath samples. Only samples in which both the ^{12}C and corresponding ^{13}C centroids were detected within ±5 ppm of the theoretical *m/z* values were considered evaluable.

(3) In the **Supplementary Information**, we replaced the original **Figure S8** with (Page S10, lines 124-131, in clean version of revised Supplemental information):

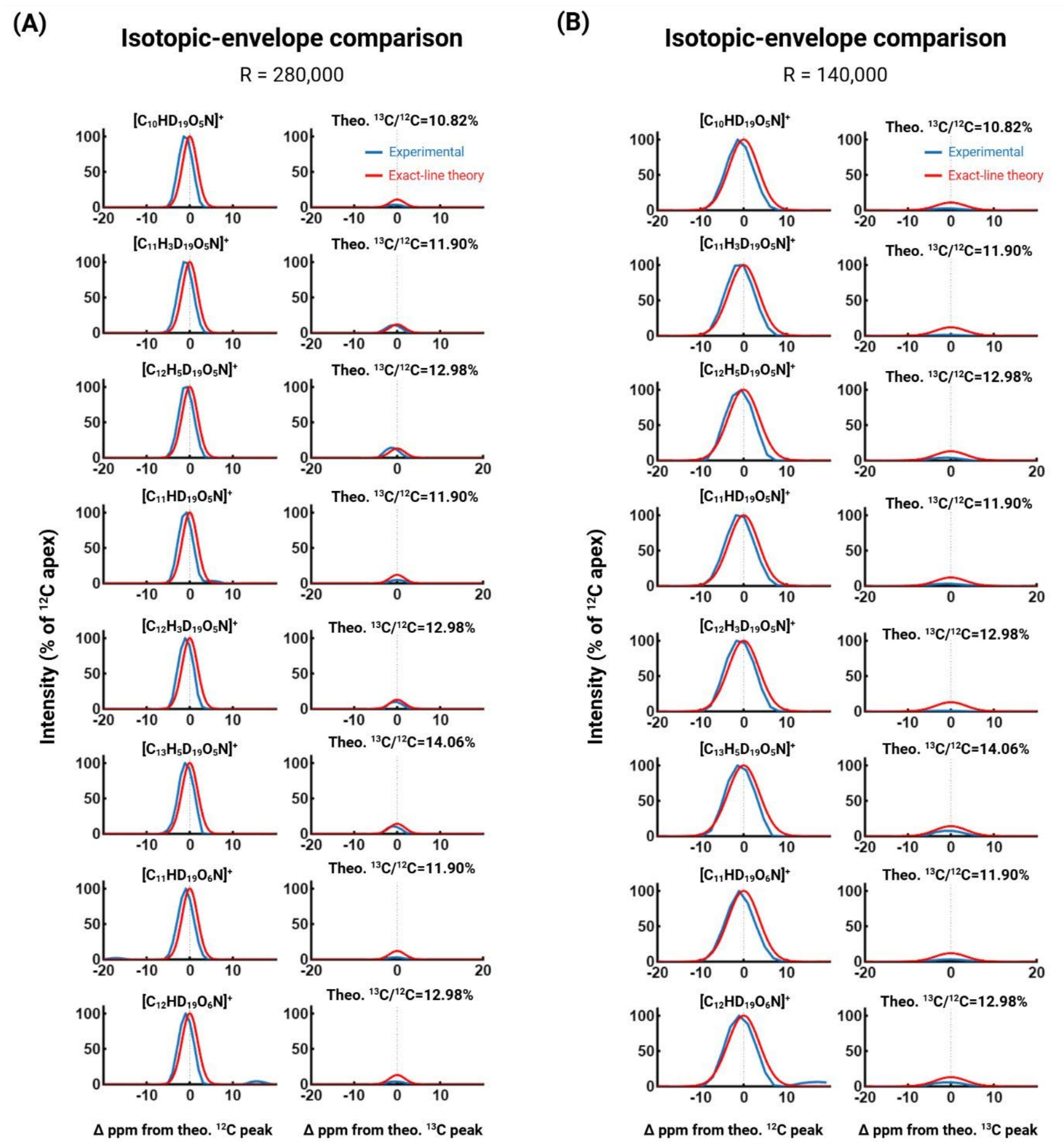

Figure S8 Isotopic-envelope comparisons for eight putative 19D-labeled acylcarnitines. (A) Representative breath samples from the Swiss discovery cohort were compared with theoretical profiles generated at a resolution of 280,000. (B) Representative breath samples from the Chinese validation cohort were compared with theoretical profiles generated at a resolution of 140,000. In both panels, comparisons are shown for the ^{12}C and corresponding ^{13}C windows, with intensities normalized to the ^{12}C apex. The eight putative 19D-labeled acylcarnitines and their theoretical exact-line isotope parameters are listed in **Table S2**.

Accordingly, we added **Table S2** to the **Supplementary Information** (Page S11, lines 142-144, in clean version of revised Supplemental information):

Table S2 Theoretical *m/z* values of monoisotopic and single-^{13}C $[M+H]^+$ peaks and expected $^{13}C/^{12}C$ intensity ratios for the eight putative 19D-labeled acylcarnitines

Elemental formula	No. of C atoms	Theo. *m/z* ($[M+H]^+$, ^{12}C)	Theo. *m/z* ($[M+H]^+$, single-^{13}C)	Theo. $^{13}C/^{12}C$ (%)
C10D19O5N	10	253.25286	254.25621	10.82
C11H2D19O5N	11	267.26851	268.27186	11.90
C12H4D19O5N	12	281.28416	282.28751	12.98
C11D19O5N	11	265.25286	266.25621	11.90
C12H2D19O5N	12	279.26851	280.27186	12.98
C13H4D19O5N	13	293.28416	294.28751	14.06
C11D19O6N	11	281.24777	282.25113	11.90
C12D19O6N	12	293.24777	294.25113	12.98

(4) In addition, a new subsection entitled **"Targeted Exact-line Isotope Validation"** was added in the **Methods** section of the supplementary information (Page S16, lines 269-285, in clean version of revised Supplemental information): "Targeted isotope validation of highly labeled features was performed using a custom exact-line model implemented in MATLAB R2025b (MathWorks Inc., USA). This analysis focused specifically on the eight putative 19D-labeled acylcarnitines summarized in **Table S2.** Elemental formulas were used to calculate the theoretical *m/z* values of the monoisotopic $[M+H]^+$ peak and the corresponding single-^{13}C isotopic peak, as well as the expected $^{13}C/^{12}C$ intensity ratio. Theoretical ^{12}C and ^{13}C peak shapes were simulated as Gaussian profiles at Orbitrap resolving powers of 280,000 and 140,000, corresponding to the two acquisition settings used in this study. Only breath samples in which both the ^{12}C and corresponding ^{13}C centroids were detected within ±5 ppm of the theoretical *m/z* values were considered evaluable. Raw profile windows centered on

the theoretical ^{12}C and ^{13}C positions (±10 ppm) were compared directly with the theoretical profiles without baseline correction, normalization, or ppm-shift optimization. The ^{12}C window, the ^{13}C window, and the combined $^{12}C+^{13}C$ window were quantified using Pearson correlation, cosine similarity, normalized root-meansquare error (NRMSE), coefficient of determination (R^2), centroid ppm error, and profile-apex ppm error."

Comment 8: *No breath blank or ambient air control is described. Since SESI-HRMS samples breath in real time, it would be important to confirm that detected D-labeled features are not artifacts from ambient deuterium-containing compounds or instrument background. Was any ambient air sampling performed?*

Response: We thank the reviewer for this comment and would like to clarify this point in more detail below. In the present study, dedicated ambient air sampling or formal blank controls were not systematically included. In our SESI-HRMS configuration, samples were actively pushed through the sample line into the SESI source rather than being passively drawn. When no sample was being introduced, a fraction of the clean gas (supplied via the auxiliary gas) continuously flowed through the ionization region and sample line and was exhausted outward through the sample inlet. Under these conditions, a direct contribution from ambient air would be less likely to account for the observed D-labeled features in this study. In addition, the D-labeled features discussed here were interpreted primarily based on their temporal D/H ratio dynamics. These features were at background level before D_2O ingestion and then showed clear time-dependent changes following D_2O administration and yogurt intake. This type of temporal pattern would be less consistent with a stable ambient or instrumental background. On this basis, although dedicated ambient air controls were not included, we consider such a background unlikely to be the main explanation for the dynamic D-labeled features reported here.

Comment 9: *The CO_2 threshold for end-tidal selection (>3%, line 346) is on the lower end — many breath studies use >4–5% to ensure true end-tidal air. The rationale for the 3% cutoff should be justified, as it may affect the metabolite profiles captured.*

Response: We fully agree that higher CO_2 thresholds (e.g., >4–5%) are more commonly used when the objective is to enrich for strictly end-tidal air. We also acknowledge that our previous wording did not adequately reflect the intended scope of the analyzed breath fraction. In particular, the statement that scans collected at CO_2 concentrations >3% represented "only the end-tidal fraction" was too strong. In this study, the >3% CO_2 threshold was applied not to define a strict end-tidal fraction, but to minimize the influence of early exhaled dead-space air without restricting the analysis to strictly end-tidal air alone, thereby preserving a broader range of metabolites associated with both airway and systemic metabolic processes.

Therefore, the rationale for applying the 3% threshold has been revised in the **Methods** section of the Supplementary Information (Page S14, lines 221-225, in clean version of revised Supplemental information): "For each breath measurement, a representative mass spectrum was generated by averaging all scans acquired during exhalation windows with CO_2 concentrations >3% in order to reduce the contribution of early exhaled dead-space air while retaining a broader range of breath metabolites.[1,3]"

Relevant references have also been provided in the revised manuscript:

(1) Singh, K. D.; Tancev, G.; Decrue, F.; Usemann, J.; Appenzeller, R.; Barreiro, P.; Jaumà, G.; Macia Santiago, M.; Vidal de Miguel, G.; Frey, U.; Sinues, P. Standardization procedures for real-time breath analysis by secondary electrospray ionization high-resolution mass spectrometry. *Anal. Bioanal. Chem.* **2019**, *411* (19), 4883-4898.

(3) Suarez-Sipmann, F.; Bohm, S. H.; Tusman, G. Volumetric capnography: the time has come. *Curr. Opin. Crit. Care* **2014**, *20* (3), 333-339.

Comment 10: *Inter-instrument variability: The two cohorts used SESI-HRMS platforms at two different sites (Switzerland and China). Were the instruments calibrated identically? Were cross-site quality control samples used? This is particularly relevant since D/H ratios are being compared across cohorts.*

Response: We appreciate the reviewer's important comment. In this study, the Swiss and Chinese cohorts were analyzed using the same standardized real-time breath analysis platform established in our previous work for multicenter studies,[2] and the same analytical configuration and acquisition settings were used at both sites to the extent feasible. In addition, all measurements were performed following the same standard operating procedures. The same quality control procedure was also applied at both sites: 100 ppb α-pinene standard gas was measured in positive mode before the first breath measurement of each day, and weekly external mass calibration was performed using the same commercial calibration solution.

We would also like to clarify that the inclusion of the second cohort (China) was primarily intended to examine whether the main temporal labeling patterns could be reproduced in an independent population at a different study site. It was not designed to support a strict quantitative comparison of D/H ratios between sites. Rather, the validation cohort served to strengthen the reproducibility of the observed temporal behavior and to support the broader applicability of the breath-tracing approach.

Accordingly, a new subsection entitled **"Instrument Settings and Quality Control"** was added in the **Methods** section of the supplementary information (Page S14, lines 197-217, in clean version of revised Supplemental information): "The Swiss and Chinese cohorts were analyzed using the standardized real-time breath analysis platform established in our previous work for multicenter studies.[2] A harmonized analytical workflow and broadly comparable acquisition settings were used at both study sites. The platform consisted of three main components: (1) an expiratory interface (Exhalion, Fossiliontech, Spain), (2) an atmospheric-pressure secondary electrospray ionization source (Super SESI, Fossiliontech, Spain), and (3) a highresolution mass spectrometer (Thermo Fisher Scientific, USA). For the Swiss

discovery cohort, a Q Exactive Plus with an enhanced resolution of 280,000 was used, and for the Chinese validation cohort, a Q Exactive with a normal resolution of 140,000 was used. The breath sample was passed through a sample line heated to 130 °C into the ionization chamber of the SESI source to minimize compound adsorption onto the tubing walls. The ionized analytes were then directly analyzed by high-resolution mass spectrometry. Mass spectrometric acquisition was performed in full-scan mode over a mass range of 70–1000, with a spray voltage of ± 2.8 kV, sheath gas flow of 60, auxiliary gas flow of 2, capillary temperature of 275 °C, and 2 microscans. During the study period, the same quality control procedure was applied at both sites: in positive mode, 100 ppb α-pinene standard gas was measured through the SESI-HRMS platform before the first daily breath measurement. In addition, weekly external mass calibration was performed using the commercial calibration solution (Pierce™ ESI Positive/Negative, Thermo Fisher Scientific, USA)."

Refences:

(2) Gisler, A.; Singh, K. D.; Zeng, J.; Osswald, M.; Awchi, M.; Decrue, F.; Schmidt, F.; Sievi, N.; Chen, X.; Usemann, J.; et al. An Interoperability Framework for Multicentric Breath Metabolomic Studies. iScience **2022**, 25 (12), 105557.

Comment 11: *The exhalation flow rate of 7 L/min (line 330) is relatively high and may not be sustainable for all participants equally, potentially introducing variability in breath dilution. How was compliance with this flow rate monitored and ensured?*

Response: We agree that inter-individual differences in exhalation performance could potentially contribute to variability in breath dilution. In the revised manuscript, we have clarified that the breath interface (Exhalion, Fossiliontech, Spain) was integrated into the breath sampling platform to assist in the control and reproducibility of the exhalation maneuver. During sampling, Exhalion provided real-time visual feedback and continuously recorded CO_2 (%), pressure drop (mbar), exhalation flow rate (L/min), and exhaled volume (L). In addition, before formal breath measurements, each participant

performed a rehearsal of the standardized breathing maneuver under the guidance of the researcher.

The following text has been now provided in the **Methods** section of the supplementary information, to better explain how exhalation performance was monitored and supported during sampling (Page S13, lines 185-192, in clean version of revised Supplemental information): "The on-line breath measurements were performed following the standardized operational procedures.[1] The breath interface (Exhalion, Fossiliontech, Spain) was integrated into the analytical platform to assist in the control and reproducibility of the exhalation maneuver. During sampling, it provided real-time visual feedback and continuously recorded CO_2 (%), pressure drop (mbar), exhalation flow rate (L/min), and exhaled volume (L). Before formal breath measurements, each participant performed a rehearsal of the standardized breathing maneuver under researcher guidance." Refences:

(1) Singh, K. D.; Tancev, G.; Decrue, F.; Usemann, J.; Appenzeller, R.; Barreiro, P.; Jaumà, G.; Macia Santiago, M.; Vidal de Miguel, G.; Frey, U.; Sinues, P. Standardization procedures for real-time breath analysis by secondary electrospray ionization high-resolution mass spectrometry. *Anal. Bioanal. Chem.* **2019**, *411* (19), 4883-4898.

Comment 12: *Fasting duration variability: Participants fasted "at least eight hours" — this means fasting duration varied between individuals, which could affect baseline metabolic state and D/H ratios. Was baseline fasting duration recorded and considered in the analysis?*

Response: We thank the reviewer for pointing this out. Participants were instructed to fast for at least eight hours before breath sampling to minimize the influence of prior food intake on baseline measurements. Beyond this minimum requirement, fasting duration was not further specified in the study protocol.

To clarify this point, the following statement has been added to the **Results and Discussion** section (Page 5, lines 118-121, in clean version of revised manuscript):

"Some inter-individual variability was observed at baseline, which may partly reflect differences in fasting status. Participants were instructed to fast for at least eight hours, but fasting duration beyond this minimum was not further specified." Furthermore, in the **Methods** section of the Supplementary Information, the original sentence "All participants were required to fast at least eight hours the night before the data collection." has been revised to (Page S12, lines 159-160, in clean version of revised Supplemental information): "All participants were instructed to fast for at least eight hours before breath sampling to minimize the influence of preceding food intake on baseline measurements."

Comment 13: *The relationship to deuterium metabolic imaging (DMI) is mentioned only via reference 3 (de Feyter et al., 2018). Given that DMI is an emerging and highprofile field, a sentence or two explicitly positioning SESI-HRMS breath analysis as a complementary or orthogonal approach to DMI could strengthen the introduction and broaden the paper's appeal.*

Response: We appreciate this suggestion and fully agree with the reviewer. In the **Introduction** section, the following text has been provided associated with reviewer's suggestion (Pages 2-3, lines 54-68, in clean version of revised manuscript): "Despite its promise, most current implementations of D-based metabolic tracing rely on blood or tissue sampling followed by liquid- or gas chromatography–mass spectrometry (LC–MS or GC–MS), which are impractical for longitudinal or real-time monitoring lengthy analytical turnaround. More recently, deuterium metabolic imaging (DMI) has further highlighted the broader potential of D-based metabolic interrogation *in vivo* by combining D-labeled substrates with magnetic resonance spectroscopic imaging to generate spatially resolved metabolic maps within tissues.[3] In parallel, real-time breath analysis has emerged as a compelling non-invasive approach for monitoring metabolic dynamics, with exhaled volatile/semi-volatile breath metabolites providing access to systemic metabolic processes.[13] Within the broader landscape, breath-based isotopic tracing may complement DMI by offering a distinct type of metabolic readout. Whereas

DMI emphasizes spatially resolved mapping of labeled metabolism within tissues, breath analysis is well suited for real-time and repeated monitoring of dynamic metabolic changes reflected in exhaled metabolites." Refences:

(3) De Feyter, H. M.; Behar, K. L.; Corbin, Z. A.; Fulbright, R. K.; Brown, P. B.; McIntyre, S.; Nixon, T. W.; Rothman, D. L.; de Graaf, R. A. Deuterium metabolic imaging (DMI) for MRI-based 3D mapping of metabolism in vivo. *Sci. Adv.* **2018**, *4* (8), eaat7314.

(13) Popov, T. A. Human exhaled breath analysis. *Ann. Allergy, Asthma, Immunol.* **2011**, *106* (6), 451456.

Comment 14: *The gut microbiome angle is touched on throughout but never fully developed. Given that SCFAs and their D-labeling patterns are explicitly linked to microbial fermentation, and that inter-individual differences are attributed to microbial variability, the absence of any microbiome characterization of the participants is a notable gap — particularly for the validation cohort in China, where gut microbiome composition is known to differ substantially from European populations. This is wort acknowledging explicitly in the limitations.*

Response: We appreciate this comment and have made the requested changes in the revised version.

The limitations stated in the **Results and Discussion** section have been revised to (Page 14, lines 338-344, in clean version of revised manuscript): "Third, metabolite annotation in the current work remains partly putative because it relies primarily on accurate-mass MS^1 data, while interpretations involving potential microbial contributions are further limited by the absence of direct microbiome characterization. Future studies combining multi-omics readouts (e.g., microbiome profiling) with systematic MS/MS validation, and confirmation with chemical standards for key metabolites, should improve annotation confidence and mechanistic insight."

In addition, the corresponding discussion **Results and Discussion** section has also been revised to use more cautious wording regarding potential microbial contributions (Page 5, lines 123-130, in clean version of revised manuscript): "Given that short chain

fatty acids (SCFAs) are primarily derived from gut microbial metabolism,[23, 24] these differences may reflect variation in gut microbial composition and metabolic activity among participants. Although yogurt intake was standardized to 300 g and the products showed broadly comparable macronutrient profiles (approximately 10% carbohydrates, 3% protein, and 10% fat, predominantly saturated), the yogurt used in the two cohorts was purchased locally. As a result, compositional differences, such as differences in microbiota, may have contributed to the variability observed between cohorts"

Refences:

(23) Macfarlane, S.; Macfarlane, G. T. Regulation of short-chain fatty acid production. *Proc. Nutr. Soc.* **2003**, *62* (1), 67-72.

(24) Dalile, B.; Van Oudenhove, L.; Vervliet, B.; Verbeke, K. The role of short-chain fatty acids in microbiota-gut-brain communication. *Nat. Rev. Gastroenterol. Hepatol.* **2019**, *16* (8), 461-478.

Comment 15: *Clinical translation pathway is underdeveloped. A slightly more explicit discussion of what disease areas or clinical questions this technology could realistically address in the near term would strengthen the impact of the Conclusions section. The diabetes/oncogenesis link via pyruvaldehyde is a good start, but could be expanded slightly.*

Response: We thank the reviewer for this valuable comment and agree with the point raised. In the revised manuscript, we therefore tightened the conclusion to better reflect the proof-of-concept nature of the present study and expanded the final discussion to more explicitly highlight application areas that are most closely aligned with our findings. In particular, we now emphasize the potential relevance of this framework for studying postprandial regulation, metabolic flexibility, host-microbial metabolic interactions, and metabolic conditions characterized by impaired postprandial control or altered carbon fluxes, such as insulin resistance and early type 2 diabetes.

Accordingly, the text in the **Conclusion** section has been carefully revised to (Page 14, lines 347-359, in clean version of revised manuscript): "In this proof-of-concept study,

we show that D_2O-based isotopic labeling combined with SESI-HRMS enables non-invasive, time-resolved assessment of metabolic dynamics through exhaled breath. High-resolution isotopic analysis revealed reproducible labeling dynamics across fasting-to-feeding transitions and linked D-H exchange features to central carbon, SCFA, and amino-acid metabolism. Together, these findings support breathbased deuterium tracing as a dynamic *in vivo* readout of nutritional and metabolic responses, with particular relevance for postprandial metabolic phenotyping, metabolic flexibility, and host-microbial metabolic interactions. With further targeted validation in larger and more diverse cohorts, this framework may provide a useful tool for investigating metabolic conditions marked by impaired postprandial control, altered carbon fluxes, or dysregulated lipid handling, particularly insulin resistance and early type 2 diabetes, and potentially related obesity-associated metabolic dysfunction."

Other minor points

Comment 16: *Table 1 is missing units for age — it lists numerical values but no "years" header, unlike weight (kg) and BMI (kg/m²).*

Response: We have added the unit "years" to the age header in **Table 1**.

Comment 17: *Line 334: "The commercially disposable plastic mouthpiece...were used" — should be "was used."*

Response: The sentence has been corrected as suggested.

Comment 18: *Line 72: The sentence "Yielding a total of 213 breath measurements" is a fragment — it should be connected to the preceding sentence with a comma or rewritten (e.g., "...except for one subject, who provided 13 measurements, yielding a total of 213 breath measurements.")*

Response: We sincerely thank the reviewer's careful review, and the sentence has been corrected accordingly to: "All participants performed 20 breath measurements (4 pre- and 16 post-D_2O ingestion), except for one subject, who provided 13 measurements (1 pre- and 12 post-D_2O ingestion), yielding a total of 213 breath measurements."

Comment 19: *Line 163: "included" should be "including" — "Other metabolites putatively annotated in this cluster likewise showed transient postprandial increases, included γ-aminobutyric acid..."*

Response: The word has been corrected as suggested.

Comment 20: *The correspondence between Figure numbers in the main text and supplementary figures (S1–S9) is dense. A brief supplementary methods section explaining what each supplementary figure shows would improve navigability for readers.*

Response: We thank the reviewer for this helpful suggestion. To improve navigability, we have added a brief Description section in the **Supplementary Information**, summarizing the content and purpose of each supplementary Figure/Table (Page S3, lines 36-50, in clean version of revised Supplemental information):

"Guide to Supplementary Figures and Tables:

Figures S1–S4 summarize the time-trace behavior of D/H ratios and the NAvMix clustering workflow, including: **Figure S1,** full-scale profiles of butyric acid D/H ratio time traces across the study cohort (N = 11 subjects); **Figure S2,** model selection and final cluster sizes for NAvMix analysis; **Figure S3,** a heat map of cluster of D/H ratio time traces behavior. **Figure S4,** time traces of 1,026 standardized D/H ratios assigned to ten clusters.

Table S1 summarizes the selected positive-response clusters used for downstream interpretation.

Figures S5–S7 show pathway enrichment analyses for Clusters #4, #7, and #10, respectively, whereas **Figure S10** presents the combined enrichment analysis across these three representative clusters.

Figure S8 Isotopic-envelope comparisons for eight putative 19D-labeled acylcarnitines. And **Table S2** lists these features and their theoretical exact-line isotope parameters."

In addition, we have included a brief introduction to the Supporting Information has been provided before the Acknowledgement Section (Page 15, lines 362-369, in clean version of revised manuscript): "Supporting Information: Supplementary results, figures, references, and experimental methods, including D/H-ratio time-trace profiles, NAvMix clustering analyses, pathway enrichment analyses, and targeted exact-line isotope validation with isotopic-envelope comparisons for putative highly labeled acylcarnitines (DOC). Supplementary Table 1 containing putative HMDB annotations for 315 isotopologue pairs from Clusters #4, #7, and #10 and Supplementary Table 2 containing enrichment statistics and ranked pathway information (XLSX)."

oc-2026-00078m.R2

Name: Peer Review Information for "In vivo real-time metabolic isotopic tracing via breath analysis upon deuterated water ingestion: a proof-of-concept study"

Second Round of Reviewer Comments

Reviewer: 1

Comments to the Author

I have carefully reviewed the author's responses and the additions; they have addressed all my concerns. I am happy with the revisions, and recommend the revised manuscript for publication.

Author's Response to Peer Review Comments:
